# Wealth Accumulation and the Gender Wealth Gap Across Couples’ Legal Statuses and Matrimonial Property Regimes in France

**DOI:** 10.1007/s10680-022-09632-5

**Published:** 2022-08-09

**Authors:** Nicolas Frémeaux, Marion Leturcq

**Affiliations:** 1 LEMMA - Université Paris 2, 4 rue Blaise Desgoffe, 75006 Paris, France; 2 Institut National d’Etudes Démographiques (INED), Campus Condorcet, 9 Cours des Humanités, CS 50004, 93322 Aubervilliers Cedex, France

**Keywords:** Wealth inequality, Gender wealth gap, Marriage, Property regime, Civil union, Unmarried cohabitation, J12, E21, D31

## Abstract

This paper examines wealth accumulation among couple-headed households and investigates changes in within-household inequality over time and across couple statuses. Going beyond previous research that mostly studies wealth accumulation within marriages by comparing married with unmarried individuals, we consider the legal statuses of couples (cohabitation, civil union, and marriage) and property regimes (community and separate property). We apply multivariate regression analysis to high-quality longitudinal data from the French wealth survey (2015–2018) and find no differences in net worth accumulation between couples’ legal statuses when property regimes are not accounted for. However, couples with a separate property regime accumulate more wealth than couples with a community property regime, and married couples with a separate property regime drive this association. Our results show that the gender wealth gap is larger for couples with a separate property regime, but it is partially compensated by accumulated wealth. Our results highlight the importance of legal statuses and property regimes in explaining the dynamics of between- and within-household inequality in France, specifically within a context of increasingly diversified marital trajectories.

**Supplementary Information:**

The online version contains supplementary material available at 10.1007/s10680-022-09632-5.

## Introduction

Wealth is an important dimension of economic well-being (Killewald, 2017; Hamoudi & Dowd, 2013; Wolff & Zacharias, 2009), and understanding its determinants has become increasingly scrutinized in the sociological, demographic, and economic literature. Although wealth inequality between households has been increasing throughout the developed world over recent decades (Piketty, 2013), a less documented phenomenon is the substantial wealth inequality within households that has been found in several countries, such as France (Frémeaux & Leturcq, 2020), Germany (Sierminska et al., 2018) or Austria (Rehm et al., 2022). It increased in France between 1998 and 2015 (Frémeaux & Leturcq, 2020), while it decreased in Germany between 2002 and 2012 (Sierminska et al., 2018).

It is now well established that marriage is an important determinant of wealth: Continuously married men and women are wealthier than never-married people or those with a disrupted marital life (Wilmoth & Koso, 2002; Zagorsky, 2005; Bonnet et al., 2022; Nutz, 2022). In terms of marital and inheritance regimes, the legal frameworks of marriage shape within-household wealth distribution and gendered patterns of wealth ownership (Deere & Doss, 2006).

The recent literature has broadened our understanding of the links between marital status and wealth inequality, but it suffers from two important shortcomings. First, the literature focuses mostly on marriage and ignores other marital statuses. By comparing strictly married to unmarried individuals, studies have analyzed how wealth is associated with marriage (Ruel & Hauser, 2013; Addo Fenaba & Lichter Daniel, 2013) and how the gender wealth gap within couples is related to marriage (Grabka et al., 2015; Meriküll et al., 2021). For example, Lersch (2017) explored how transitioning into marriage affects wealth by following individuals over time and considering gendered differences, while Kapelle & Lersch (2020) studied how individuals accumulate wealth within marriage. Wealth accumulation during marriage is associated with pre-marriage history, as couples who have cohabited before marriage are wealthier than those who married directly (Painter & Vespa, 2012). However, cohabitation is increasingly forming a part of long-term marital status (Lesthaeghe, 2020), and couples start accumulating wealth while cohabiting. The previous research has considered unmarried cohabitation as a transition to marriage and ignored out-of-wedlock wealth accumulation. Thus, the second shortcoming is that the recent literature has failed to consider the new types of legal statuses established by some countries such as France (the country under study in this paper), Belgium, and the Netherlands. By contracting a civil union or registered partnership, partners gain some legal recognition without being married. To our knowledge, the association between registered partnership and wealth has not been analyzed in the literature.

Very little of the literature has explored the association between wealth and property regimes, and it has done so by comparing couples in different property regimes at a given point in time. This literature suggests that community property regimes protect against large intrahousehold gender wealth inequality. In France, married couples with a separate property regime have been found to be richer than married couples with a community property regime (Frémeaux & Leturcq, 2013), and the within-couple gender wealth gap is larger among couples with separate rather than community property regimes (Frémeaux & Leturcq, 2020). Comparing three developing countries, Deere et al. (2013) show that the gender wealth gap is larger in Ghana and India (where marriage imposes a separate property regime) than in Ecuador (with a partial community property regime).

These studies mix initial levels of wealth and changes over time. Regarding household wealth accumulation among married couples, greater wealth is found among those with separate rather than community property regimes; but it is unclear if wealthier couples opt for a separate property regime or if couples with a separate property regime accumulate more wealth over time while married. Similar doubts exist concerning the gender wealth gap: although couples with a separate property regime exhibit a higher gender wealth gap, it is unclear if those with an initial wealth gap decide to separate their assets (as observed in France; Frémeaux & Leturcq, 2013) or if married partners with a separate property regime accumulate wealth at different paces within households.

The goal of this paper is to fill the gaps in our understanding of how the legal forms of couples contribute to the emergence of between- and within-household inequality. Here, we address two research questions: (i) Does wealth accumulation differ across couples’ legal statuses, property regimes, and the different combinations of both? and (ii) Do changes in the intrahousehold gender wealth gap over time differ across couples’ legal statuses and property regimes?

To analyze wealth accumulation and changes in the intrahousehold gender wealth gap over time and across couples’ legal statuses and property regimes, a specific context and precise data are required. More precisely, we need a national context with a variety of well-defined marital statuses and property regimes that can be observed in the data. This is not the case in countries where, for instance, unmarried cohabitation is not an option for couples or they cannot sign prenuptial agreements. Moreover, analyzing the intrahousehold gender wealth gap requires data that allow observing personal wealth—that is, data that gather wealth information at the individual level within the household. Because most surveys and administrative data collect information about wealth at the household level, using it to analyze wealth at the individual level requires making assumptions about the intrahousehold distribution of assets. Lastly, analyzing changes over time requires observing the same individuals over time in longitudinal data.

France provides a unique case for observing wealth accumulation and the intrahousehold gender wealth gap across couples’ legal statuses and property regimes, due to its diverse legal statuses (marriage, civil union, and unmarried cohabitation) and property regimes (community and separate property regimes). We use the first two waves of the longitudinal component of the French wealth survey (INSEE-*Enquête Histoire de Vie et Patrimoine*), collected in 2014–2015 and 2017–2018. The French wealth survey follows roughly 6,000 households over time and provides high-quality data on personal wealth at the individual level within the household (real estate, financial and business assets, and liabilities). It also provides precise information on the marital status of individuals, thus allowing us to determine the legal statuses of couples (unmarried cohabitation, civil union, or marriage) and their property regimes (community property regime or separate property regime). The French wealth Survey is unique among international sources of information for understanding wealth accumulation and the gender wealth gap across couples, as it combines longitudinal information on the personal wealth of individuals within couples together with precise information on their marital statuses.

We use multivariate regression models to examine couples’ wealth accumulation and its contribution to the within-couple wealth gap between 2015 and 2018, according to their legal statuses and property regimes. In focusing on longitudinal changes in wealth across couples, we thus compare wealth accumulation across couples and not differences in their initial level of wealth. We define wealth accumulation as the difference in value of the net household wealth between 2015 and 2018. Our analysis makes no claim to having a causal interpretation, as couples self-select into marital status and property regime. By including controls for potential confounding factors, we are able to understand the links between couple statuses and wealth.

The remainder of the paper is organized as follows. Section 2 describes the French context. In Sect. 3, we present the theoretical background and formulate the hypotheses. Sect. 4 presents the data and descriptive statistics. Section 5 provides the multivariate analysis. Section 6 concludes.

## Context

### Legal Context

Couples in France can obtain three legal statuses: unmarried cohabitation,^1^ civil union (called PACS^2^), and marriage. Married and PACSed couples can choose from a menu of matrimonial property regimes, which can be broadly classified into two main systems:

1. *Community property regime*^3^: All assets and debts accumulated during the marriage (or PACS) are jointly owned by the spouses, as long as these assets are not inherited. Assets acquired before marriage (or PACS) and through inheritance remain individual assets. Returns on individual assets are considered joint property. In case of separation, spouses must equally share all joint assets, even if they contributed unequally to their acquisition. This is the default regime for married couples.
2. *Separate property regime:* Couples legally hold all their assets and returns on their assets separately. This regime excludes redistribution within the household, because each asset belongs to the spouse who acquired it. Some assets may be held jointly, such as the main residence. Opting for a separate property regime comes at some moderate cost for married couples, because the spouses must sign a prenuptial agreement at a notary’s office before marriage.

Unmarried partners are legally considered as two separate individuals and are thus *de facto* subject to a separate property regime in terms of asset ownership. Any asset they acquire is presumably held as an individual asset, but they can define a joint property title when they jointly acquire real estate.

The community of acquisitions regime has been the default regime for married couples since 1965 and for PACSed couples from 1999 to 2006. From 2007 onward, the separate property regime has been the default regime for PACSed couples, although they can easily opt for a different regime when signing their PACS contract.

Table 1 presents some important legal features of marriage, PACS, and unmarried cohabitation. PACS is close to marriage in most aspects, such as taxation on income and inheritance, social rights, and the obligations of partners. However, it is similar to unmarried cohabitation in terms of acquiring citizenship, adopting children, and separation. PACS is easy to dissolve and can be done so unilaterally, while, as unmarried couples, PACSed couples cannot petition for financial compensation upon separation.

### Changes in the Couple Statuses Over Time

The marital landscape in France has been radically transformed over recent decades.

The share of cohabiting couples among all couples increased from 3% in 1962 (Daguet, 1996) to 19% in 2015 (Costemalle, 2017). Between 2011 and 2014, roughly 546,000 cohabiting couples formed yearly—approximately twice as many as marriages (Costemalle, 2017). During the 2015–2018 period of data collection, unmarried cohabitation was the most frequent way to start a formal union.

PACS have become popular in France, with the annual number of these civil unions rising steeply in the 2000s and reaching a plateau in the 2010s. In 2018, 209,000 PACS were contracted, which is slightly lower than the 228,000 marriages that same year. Despite its popularity, only 7% of all couples were PACSed in 2018 because it was only recently created and also may be used as a transitory status before marriage.

Approximately 230,000 marriages were registered yearly in France during the 2010s. This seeming stability hides the less documented change of a growing share of newlywed couples opting out of the default property regime and into the separate property regime. Approximately 20% of newlywed couples chose the separate property regime in 2010-up from less than 10% in the 1970s (Frémeaux & Leturcq, 2018).

The transformation of the marital landscape over the past 50 years in France has had implications for household wealth. Although the most common marital status of couples remains marriage with a community property regime, it is no longer the overwhelming status it used to be. This—partial—withdrawal from marriage with a community property regime has led to individualized wealth among couples and an increase in the within-couple wealth gap (Frémeaux & Leturcq, 2020).

## Theoretical Background and Hypotheses

### Theoretical Framework

#### Wealth Accumulation Across Couple Statuses

In a first analysis, we study the wealth accumulation process at the couple level. The couple’s wealth comprises the female partner’s personal wealth, the male partner’s personal wealth, and joint assets. We consider these assets together to construct $W_{it}$, which represents wealth of couple *i* at beginning of period *t*. Standard economic models suggest that wealth accumulation over a given period *t*, written as $W_{it+1}-W_{it}$, follows the dynamic:

1 $$\begin{aligned} W_{it+1}-W_{it} = r_{it} W_{it} + S_{it} + B_{it} \end{aligned}$$

where $r_{it}$ is a composite rate of return on wealth for couple *i*, and it depends on the wealth portfolio of couple *i*^4^. $S_{it}$ represents the savings of couple *i* during period *t* (potentially negative), and $B_{it}$ describes wealth transfers (bequests, donations, or windfall wealth such as lottery wins) received or given by the couple.

Equation (1) is an accounting equation describing the components of wealth accumulation: initial wealth $W_{it}$ generates additional wealth at a rate of return $r_{it}$; savings $S_{it}$ and bequests $B_{it}$ add to (or subtract from) the stock of wealth. Each component is related to the economic and socio-demographic characteristics of individuals. We expect differences in both wealth *accumulation* and *levels* across couples.

We expect savings to differ across couple types ($S_{it}$ in Eq. 1), for which we list below seven arguments supporting this assumption.

(i) *Specialization*: A community property regime implies equal sharing of assets accumulated during marriage, thus protecting the spouse who specialized in domestic work (Cigno, 2012; Frémeaux & Leturcq, 2013; Bayot & Voena, 2015). Married and PACSed couples with a community property regime are more likely than those with a separate property regime to specialize (one member in the labor market, while the other is dedicated to domestic work). Within-household specialization may lead to lower earnings and less accumulation of wealth.
(ii) *Insurance:* The legal differences across couple statuses listed in Table 1 induce more insurance for couples who are married than for those who are PACSed or cohabiting, such as financial compensation after separation and a survivor pension in case of a spouse’s death. Married couples need less precautionary savings than unmarried cohabitants or PACSed couples.
(iii) *Precautionary savings:* When the cost of separation is low, couples anticipate a higher risk of separation and set aside more precautionary savings (Angelini et al., 2019; González & Özcan, 2013). Because, in the French context, the cost of separation is lower for unmarried cohabitation and PACS than for married couples, they should set aside more precautionary savings than married couples. Regarding matrimonial property regimes, the literature has shown that when the cost of divorce decreased in the USA due to the introduction of unilateral divorce, this led to an increase in household savings in states imposing equal division of property but not in states with separate property regimes (Voena, 2015). If similar saving behaviors are to be found in the French context, we expect PACSed couples with a community property regime to maintain more savings than married couples with the same regime, while no difference should exist between married and PACSed couples with separate property regimes.
(iv) *Incentive*: Matrimonial property regimes induce different incentives to save, as a separate property regime creates individual incentives for spouses to accumulate more for themselves by reducing the share of joint accumulated wealth.
(v) *Time horizon*: Individuals involved in a relationship save more than singles because they have a longer financial planning horizon (Fulda & Lersch, 2018), and time preferences have a direct effect on savings (de Rubio, 2015; Ersner-Hershfield et al., 2009; Fisher & Montalto, 2010). The same reasoning may apply to the couple’s legal status. It has been shown that marriage serves as a commitment device for couples (Matouschek & Rasul, 2008; Cigno, 2012), due to the separation cost being higher for marriage than for unmarried cohabitation. Thus, greater commitment could be linked to a longer financial planning horizon. Because separation costs in France are the highest for marriage, the lowest for cohabitation, and intermediate for PACS, we expect married couples to have a longer planning horizon (and thus accumulate more wealth) than PACSed couples, who should have a longer planning horizon (and thus also accumulate more wealth) than cohabiting couples.
(vi) *Taxation*: In France, joint taxation of labor income (available for PACSed and married couples) generates greater gains than individual taxation (imposed on cohabiting couples) and thereby increases the saving capacity of married and PACSed couples. The gains generated by joint taxation are higher when the intrahousehold income gap is large, and almost zero when spouses have similar income (Allègre et al., 2021). Therefore, we expect the gains from taxation to be larger for married couples with a community property regime, because they are more likely to specialize than couples in unmarried cohabitation, PACS, and marriage with a separate property regime.
(vii) *Fertility and bequest*: Parents are likely to save more than non-parents because they aim to leave a bequest for their children. In many countries, couples with children are more likely to formalize their couple formation due to social and religious norms. We expect formalized couples to save more than non-formalized couples.^5^

The composite rate of return $r_{it}$ depends on the rate of return on each type of asset and on the couple’s wealth portfolio. Large variations in the rates of return across assets have been observed in France over the past decade. Garbinti et al. (2021) showed that the average annual rates of returns during the 1970–2014 period were 6.1% for housing assets, 3.7% for life insurance, and −0.2% for deposits. Therefore, the composite rate of return would be higher for couples owning a larger share of the most dynamic assets^6^ (i.e., housing assets rather than deposits and life insurance). We expect rates of return on wealth ($r_{it}$ in eq. 1) to differ across couple types due to their different wealth portfolios.

(i) *Selection into couple status:* The economic literature has shown that richer households have higher rates of return on their wealth compared to poorer households (Bach et al., 2020) because their wealth portfolios are more dynamic. In France, rich couples are more likely to marry and to choose a separate property regime at marriage (Frémeaux & Leturcq, 2013). Therefore, we expect married couples with a separate property regime to hold more dynamic assets and have higher rates of return on their wealth than other types of couples.
(ii) *Reverse causality:* A direct association between the couples’ legal status and wealth portfolio is observed if the decision to PACS or marry is related to their wealth portfolio, such as entering homeownership. In France, it has been shown that entering a PACS or marriage is closely related to buying a home (Belliot & Rebière, 2016), which may be related to these couples having better access to mortgages (Leturcq, 2014). This reverse causality explains differences in wealth portfolios across couple types, which imply differences in the rates of return on wealth across couple types.
(iii) *Cost of separation*: A wealth portfolio is associated with the cost of separation and wealth transfers induced by property regimes upon separation. In the USA, the introduction of unilateral divorce decreased the cost of divorce, which in turn reduced the likelihood of buying a home among couples living in states with a default separate property regime, while it increased in states with a default community property regime (Stevenson, 2007). In France, the cost of separation is lower for PACS and unmarried cohabitation than for marriage. If the results observed in the USA apply in France, we should observe that, among couples in a separate property regime, the likelihood of buying a home is higher among couples facing a larger cost of separation (married couples) than among couples facing a smaller cost of separation (PACS or cohabiting couples). In contrast, we should observe that, among couples in a community property regime, the likelihood of buying a home is lower among couples facing a larger cost of separation (married couples) than among couples facing a smaller cost of separation (PACS or cohabiting couples).

We expect the propensity to receive or give a bequest ($B_{it}$ in Eq. 1) to differ across couple types.

(i) *Family background*: Much of the literature has demonstrated the intergenerational transmission of wealth, showing that richer individuals are more likely to have richer parents and receive bequests from them (Hansen & Toft, 2021; Boserup, 2018; Adermon et al., 2018). In France, richer couples are more likely to opt for marriage with a separate property regime (Frémeaux & Leturcq, 2013); so, after controlling for age, married couples with a separate property regime are more likely than other couples to receive (or give) bequests.

#### Gender Wealth Gap Across Couple Statuses

In a second analysis, we study how the distribution of wealth within the household changes across time and how it differs across couple statuses. To investigate this issue, we analyze changes in the female partner’s share of household wealth over time and across couple statuses. A female partner’s wealth share of 50% indicates no wealth gap within the household, and a female partner’s share lower (resp. larger) than 50% indicates that the female partner is poorer (resp. richer) than the male partner. Wealth may not be equally distributed between partners, and even married and PACSed partners with a community property regime often hold personal assets that were acquired before their marriage or PACS, or because they were received as bequests. The female partner’s share of household wealth may change over time, even when couples accumulate joint wealth only: As the value of joint wealth increases, the female partner’s share of household wealth gets closer to 50%^7^.

In Appendix 1, we show that changes in the female partner’s share of household wealth are related to how women compare to men in terms of three components, which we expect to differ across couple types.

1. *Wealth portfolio*: If the female partner’s wealth portfolio is more dynamic than the household’s portfolio, her share of the household wealth increases. The rate of return on the female partner’s wealth differs from the rate of return on the household wealth once she (or her partner) owns some personal assets. The rate of return on the female partner’s wealth compared to the rate of return on the household wealth is likely to differ across couple types because of different wealth portfolios and because of the above-mentioned *reverse causality* assumption. Even when couples separate their assets, their primary home is often a joint asset (Frémeaux & Leturcq, 2020) and personal assets are more likely to be financial. Over the recent period, housing has been the most dynamic asset in a household portfolio, which means that joint assets (usually housing) are more dynamic than personal assets. This dynamic nature means that partners’ share of household wealth should converge toward 50% (or remain stable at around 50%). It also means that the poorer partner’s (usually the woman’s) relative share of wealth should increase, by which we expect the female partner’s share of household wealth to increase more for unequal couples than for initially equal couples. If wealth is unequally distributed between partners when they meet, couples are more likely to opt for a separate property regime (Frémeaux & Leturcq, 2013). We expect the female partner’s share of household wealth to increase more for couples with a separate property regime than for couples with a community property regime. In addition, the *reverse causality* assumption states that partners formalize their relationship when buying a home together; so we expect couples to hold more joint assets when they are married or PACSed than when cohabiting. Therefore, we expect the female partner’s share of household wealth to increase more for married or PACSed couples with a separate property regime than for unregistered cohabiting couples.
2. *Female partner’s share of savings*: The female partner’s share of the household wealth increases if her share of acquired assets (through savings on income) is larger than her initial share of the household wealth. To state it simply, if the female partner has 30% of the initial wealth and 50% of the household’s acquired assets, then her share of the household’s wealth increases. This component is directly affected by the property regime, as community regimes impose that acquired assets are shared equally within the household, whereas separate property regimes state that all acquired assets are kept as personal assets. Therefore, for married and PACSed couples with a community property regime, the direction of change in the female partner’s share of household wealth depends on which partner is initially poorer. If she is poorer than her partner, equally sharing acquired assets (i.e., 1/2 of the acquired assets) means that she gets more than her initial share (which is lower than 1/2), meaning that her share of household wealth increases. This is independent of how much each partner contributes to the household’s savings and thus independent of specialization in the household. As most women are poorer than their partners, we expect the female partner’s share of household wealth to increase for women in a community property regime. For couples with a separate property regime, the female partner’s share increases if her contribution to the household savings is larger than her initial share of household wealth. Thus, the direction of the female partner’s share of household wealth is more complicated to derive, as it depends on how the female partner’s share of household *income* compares with her initial share of household wealth.
3. *Female partner’s share in received bequest*: The female partner’s share of the household wealth increases if she receives a relatively larger bequest than her partner. There should be no pervasive intrahousehold gender differences in the likelihood of receiving a bequest. Recent research has shown that assortative matching occurs with parental wealth (Wagner et al., 2020) and with inherited wealth (Fremeaux, 2014). Qualitative evidence has shown that women receive lower bequests than men within families (Bessière & Gollac, 2020); but, to our knowledge, no empirical work has studied within-household gender differences in wealth received as bequests.

### Hypotheses

From the theoretical framework presented in Sect. 3.1, we derive four sets of hypotheses regarding the associations between: (1) legal status and wealth accumulation; (2) property regime and wealth accumulation; (3) precise marital status (marital status$\times$property regime) and wealth accumulation; and (4) precise marital status and the female partner’s share of household wealth. A summary of the hypotheses is presented in Table 2.

Our theoretical framework gives contradictory insights into the expected association between the couple’s legal status and wealth accumulation. We thus derive two competing hypotheses and test one against the other. From the *precautionary savings* and *insurance* arguments, we expect unregistered cohabiting couples to accumulate more wealth between *t* and $t+1$ than PACSed and married couples (Hypothesis H1a-i), and PACSed couples to accumulate more wealth than married couples (Hypothesis H1a-ii). We expect Hypotheses H1a-i and H1a-ii to be driven by financial wealth rather than housing wealth, as financial wealth is more easily available for consumption smoothing. On the other hand, from the *time horizon*, *taxation*, *reverse causality*, and *fertility* arguments, we expect married and PACSed couples to accumulate more wealth than unregistered cohabiting couples (Hypothesis H1b). We expect Hypothesis H1b to be driven by housing wealth, as the reverse causality argument directly links home purchasing to the legal status of couples.

Our theoretical framework also gives contradictory insights into the association between the couple’s property regime and wealth accumulation, so we derive two competing hypotheses. The *specialization* and *incentive* arguments suggest that couples with a separate property regime accumulate more wealth than couples with a community property regime (Hypothesis H2a-i). The *specialization* argument additionally suggests that controlling for household income wipes out the wealth accumulation gap between separate and community property regimes (Hypothesis H2a-ii). The *taxation* argument goes in the other direction: For a given household income, couples with a community property regime accumulate more wealth than couples with a separate property regime (Hypothesis H2b).

Our theoretical framework gives clearer insights into the association between the couple’s precise marital status and wealth accumulation. The *selection* and *family background* arguments suggest that married couples with a separate property regime accumulate more wealth than any other type of couple (Hypothesis H3a-i) because they are initially richer than any other type of couple. Controlling for initial level of wealth attenuates the wealth premium of married couples with a separate property regime (Hypothesis H3a-ii). The *cost of separation* argument indicates that married couples with a separate property regime accumulate more wealth than unregistered cohabiting couples and PACS couples with a separate property regime (Hypothesis H3b). The *cost of separation* and *insurance* arguments state that PACSed couples with a community property regime accumulate more wealth than married couples with the same regime (Hypothesis H3c). These arguments do not lead to any clear hypothesis on which type of asset drives the association between wealth accumulation and precise marital status.

Regarding the association between changes in the female partner’s share of household wealth and marital status, we expect her share to increase more with a separate rather than community property regime (Hypothesis H4a). More specifically, we expect the female partner’s share of household wealth to increase when: (i) the female partner’s share of household income is larger than her share of wealth under a separate property regime in *t* (Hypothesis H4b-i); and (ii) the female partner’s share of household wealth is lower than 1/2 under a community property regime in *t* (Hypothesis H4b-ii).

## Data

### Description of the Survey

We use the longitudinal data from the French wealth survey (*Enquête Histoire de Vie et Patrimoine*), conducted by INSEE. It is a rotating panel survey of over 6,000 single-headed and couple-headed households that are representative of the French population. Two waves are available as of the present: The first wave was collected in 2014-2015 and the second in 2017-2018^8^.

Of the 6,000 households initially included in the sample, 4,409 were surveyed in 2018 and 1,672 of them are single-headed households (singles, divorcees and widows) in at least one wave. Removing single-headed households, we started with a sample of 2,737 couple-headed households observed in both waves, to which we applied sample restrictions. First, we restricted our sample to opposite-sex couples because the survey is not well-designed for studying same-sex couples and only 28 same-sex couples are observed in both waves (2,709 observations left). Second, because this paper aims to study the wealth accumulation process and including individuals aged 60 or more would imply focusing on people who may be dis-accumulating wealth, we therefore restricted the sample to opposite-sex couples in which both spouses/partners are between 25 and 60 years old in 2015 (1,801 observations left). Third, we excluded couple-headed households experiencing marital transitions between waves,^9^ namely those transitioning from cohabitation to PACS or to marriage, or from PACS to marriage (1,684 observations left). Fourth, we excluded observations with missing values for wealth, marital status, or any other relevant characteristics,^10^ thus reducing our analytical sample to 1,666 households.

Because wealthy neighborhoods are oversampled in the French wealth survey, we used sample weights to ensure the representativeness of the French population.

### Variables

The French wealth survey provides high-quality data on personal wealth and it takes an asset perspective by asking households to describe all their assets (deposits, real estate, financial, and business) and liabilities. For each asset, households provide a short description, a self-reported value,^11^ and the identity of the household member who owns it. When an asset is held by more than one person (as are most real estate and business assets), the survey provides a detailed description of the share held by the household head, that person’s spouse or partner, other members of the household, and other persons outside the household. This precise description of assets enables us to measure wealth at the household and individual levels. The longitudinal component enables us to track how households, people, and their wealth change over time.

Our main outcome is household wealth net of liabilities, constructed as the sum of all real estate and financial assets minus liabilities.^12^ We provide additional estimates for gross household wealth in the online Appendix. The gap between net household wealth and gross household wealth is due mostly to indebted couples purchasing a home with a bank loan. We therefore expect the gap between net and gross wealth to be larger for young couples. However, even after controlling for age and birth cohort, the couples’ ability to obtain a mortgage reflects a financial potential that may only be partially ascribed to current household income. Therefore, some couples may seem poorer than other similar couples in terms of net wealth because they are indebted, but they have greater financial potential that is captured better by gross household wealth (Hansen and Toft, 2021).

Household wealth (both net and gross) is adjusted for inflation and is top- and bottom-coded at the 0.1% level (winsorization). Wealth has a heavily right-skewed distribution due to substantial wealth inequality in the population, and it includes zero and negative values. To reduce skewness, we apply an IHS transformation to household net wealth. IHS can be applied to zero and negative values, and it allows for similar interpretation of the regression results as a log transformation. The IHS transformation has been used in various applications, including wealth analysis (Friedline et al., 2015; Pence, 2006). A serious flaw to IHS transformation is that estimates based on IHS-transformed variables are sensitive to the unit of measurement (Aihounton & Henningsen, 2020; Bellemare Marc and Wichman Casey, 2020). We tested different units of measurement for wealth by following the procedure suggested by Aihounton & Henningsen (2020), and we opted for net wealth expressed in thousands of euros before applying the IHS transformation.^13^

The French wealth survey provides key information on the marital status of couple-headed households, namely legal status (unmarried cohabitation, PACS, or marriage) and property regime (community or separate property regime). No other data source provides such detailed information on property regimes in France.

The survey also includes a detailed biography of the household, its members, and its characteristics, which we use as control variables. Some of these characteristics are time-invariant: birth cohort, age difference between partners, year of couple formation, having divorced in the past, educational attainment of both partners, and being French or not.^14^ We control for the values observed in 2015. Other characteristics are time-varying: bequests and inter-vivos gifts received and given, number of children in the household, household income, employment status, self-employment status, and size of the city of residence. We control for the values in 2015 and for the variables’ changes in value between 2015 and 2018.

Controlling for time-invariant characteristics enables us to control for different couples’ selection into marital status. Birth cohort and year of couple formation allow us to consider changes in the availability of legal options (PACS) and the dynamics in wealth accumulation at different moments in the life-cycle. Age difference and having divorced in the past account for different dynamics across partners within the same couple. Citizenship controls for migration background, as migrants typically have lower net worth than natives (Rehm et al., 2022). Educational attainment allows controlling for different financial potential. Controlling for time-varying characteristics enables us to test the relevance of the mechanisms listed in Sect. 3.1. For instance, controlling for income and for employment status allows us to test for *specialization* and *taxation* mechanisms. Controlling for number of children, we will study the mechanism stating that parents are incentivize to save in order to leave a bequest to their children. Controlling for bequests and gifts that are received or given, we will study whether differences across couple types come from a different propensity to receive (and to give) bequests and inter-vivos gifts.

### Descriptive Statistics

Tables 3 and 4 provide detailed descriptions of our sample of couples. Precisely 73.7% of all couples are married: 65.8% with a community property regime and 7.9% with a separate property regime. Thus, marriage with a community property regime is the most common form of union, despite the significant changes in the marital landscape described in Sect. 2.2. Next, 16.7% of all couples are unmarried cohabitants and 9.5% are PACS, which can be further broken down to 1.9% PACSed couples with a community property regime and 7.6% with a separate property regime. These proportions are in line with Costemalle (2017), whose estimates are based on a much larger sample of French households.

Table 4 shows that the marital legal status is associated with household net wealth, with married couples being more affluent than PACSed couples, and cohabiting couples being the poorest. However, there is no clear pattern regarding property regimes, as married couples are more affluent with a separate property regime than with a community property regime while, conversely, PACSed couples are less affluent with a separate property regime than with a community property regime. Wealth accumulation between 2015 and 2018 has been greater for unmarried couples (+24%) than for PACS couples (roughly +15% for PACS with either a separate or community property regime) and for married couples with a separate property regime (+20%). The lowest is found for married couples with a community property regime (+10%).

The gender wealth gap’s association seems to be stronger with the property regime than with marital legal status. The female partner’s share of wealth for both married and PACSed couples with a community property regime is close to 50% and is stable over time. It is close to 41-42% for both married and PACSed couples with a separate property regime in 2015, and it remains stable over time for PACSed couples but increases for married couples. Among unmarried cohabitants, the female partner’s share of household wealth has also increased (from 48% in 2015 to 51% in 2018).

Table 3 also shows how demographic and socio-economic characteristics differ across marital statuses. Married couples are older, and the age gap between married spouses is larger than for PACSed couples and unmarried cohabitants. Regarding the marital history of couples, spouses in a married couple with a community property regime are less likely to have experienced a divorce than other types of couples, and their relationship has lasted longer than for other couples. Men and women in unmarried cohabitation or in a marriage with a community property regime have lower educational levels, lower employment rates, and lower household income than PACSed and married couples with a separate property regime, suggesting that opting out of the most traditional legal status is correlated with more favorable educational level and labor market outcomes. The gender employment gap is the lowest among PACSed couples, which may be related to PACSed couples having higher educational attainment or less intrahousehold specialization when compared to married couples and unmarried cohabitants as observed by Kandil & Périvier (2021). Men and women in a marriage with a separate property regime are more likely to be self-employed than men and women in any other status.^15^ The proportion of couples having received a bequest over the period is similar for unmarried cohabitants and married couples (roughly 7%) but lower among PACSed couples.

## Empirical Analysis

### Specification

In order to test the hypotheses presented in Sect. 3.2, we estimate the following model:

$$\begin{aligned} \Delta _t W_{i} = \beta _0 + \sum _k \beta _{k} {\mathbbm {1}}\{couple_{i}=k\} + \gamma ' X_{it} + \eta ' \Delta _t X_{i} + u_{i} \end{aligned}$$

In all specifications, the dependent variable $\Delta _t W_{it}$ is the change in net household wealth (expressed in thousands of euros, IHS-transformed) of couple *i* between 2015 and 2018. The coefficients $\beta _{k}$ are our coefficients of interest: They measure wealth accumulation between 2015 and 2018 for couples of type *k* relative to the reference status. We measure wealth accumulation as a difference in IHS-transformed wealth, so our left-hand side variable must be interpreted as the growth rate of wealth over the observed period. As a consequence, a positive (resp. negative) $\beta _{k}$ indicates if the wealth of couples of type *k* increased faster (resp. slower) than the reference couple type.^16^

We consider three measures of wealth: net household wealth, and its two components—net financial wealth and net housing wealth. Net household wealth is the sum of net financial wealth and net housing wealth, but the IHS-transformed net household wealth does not equal the sum of the IHS-transformed net financial wealth and IHS-transformed net housing wealth. Therefore, there is no obvious link between the $\beta _k$ obtained from separate regressions on the different components of wealth.

The definition of couple status *k* depends on which hypothesis is tested. In order to test Hypothesis 1, which links wealth accumulation to legal status, we define couple status *k* as either unmarried cohabitation, PACS, or marriage. To test Hypothesis 2, which links wealth accumulation to property regimes, we define couple status *k* as either community property regime (PACS or marriage) or separate property regime (unmarried cohabitation, PACS, or marriage). Hypothesis 3 links wealth accumulation to both legal status and property regime, and we consider couple status *k* to be unmarried cohabitation, PACS with a community regime, PACS with a separate property regime, marriage with a community regime, and marriage with a separate property regime. Hypothesis 4 links the female partner’s share of household assets to couple status, and we estimate the same model but use the change in female partner’s share of household assets as the dependent variable.

We progressively include three sets of control variables $X_{it}$, which were measured in 2015. The first are age-related and socio-demographic variables: birth cohort of the male partner (bef. 1940, 1940–1949, 1950–1959, 1960–1969, 1970–1979, 1980–1989, or 1990–1995); age gap between partners (F-M: lower than −10, [−10; −3], [−3; 2], more than 2); year of couple formation (bef. 1979, 1980–1989, 1990–1999, 2000–2009, 2010–2014); nationality of both partners (French, foreign-born French, or foreigner); and having divorced in the past (dummy). The second set comprises different socio-demographic variables: education of both partners (ISCED 0–2, ISCED 3–4, ISCED 5–8); household income (log); employment status (dummy); and self-employment status (dummy). Finally, the third set contains bequest motives: bequests and inter-vivos gifts received or given over the 2015–2018 period (dummy); and number of children in the household (none, 1, 2, 3 or more). For time-varying variables, we also control for change in time $\Delta _t X_{i}$ (between 2015 and 2018): change in log household income, change in self-employed status, and change in employment status, while also using an indicator for a child leaving the household or the birth of a child. We additionally control for the size of the city of residence.

All regressions are weighted using sample weights.

### Results

#### Wealth Accumulation

Table 5, panel A, presents the results regarding wealth accumulation across legal status, thus providing an empirical test of our first set of hypotheses. Column 1 presents estimates without any control variables. When we consider household net wealth, we observe that PACSed and unregistered cohabiting couples accumulate wealth faster than married couples between 2015 and 2018, but the difference is not statistically significant. Younger people accumulate wealth faster than older people, and they are less likely to be married: Controlling for birth cohort and year of couple formation reverses the sign of the association between legal status and wealth accumulation, thus showing a negative (but not statistically significant) association between unregistered cohabitation or PACS and wealth accumulation (column 2). The point estimates do not significantly change by adding controls for educational attainment; income and labor market status (column 3); number of children; or having received or given inter-vivos gifts or bequests (column 4). Decomposing by type of asset gives a rather different picture. We do not observe significant differences in wealth accumulation between married, PACS, and unregistered cohabiting couples in terms of housing wealth (column 5), but our results show that PACSed couples have accumulated statistically more financial wealth between 2015 and 2018 than married couples (column 6). They also indicate more wealth accumulation for unregistered cohabitants than for married couples, although the difference is not statistically significant. Our results indicate that, overall, all couples accumulate wealth at a similar pace, thus invalidating the hypothesis stating that cohabiting couples accumulate more wealth than PACSed or married couples (H1a-i) and that PACSed couples accumulate more wealth than married couples (H1a-ii). This also invalidates the opposing hypothesis, which states that PACSed and married couples accumulate more wealth than cohabiting couples (H1b). Yet, our results also show that PACSed couples accumulated more financial wealth between 2015 and 2018 than married couples, which validates our Hypothesis H1a-ii, but only for financial wealth.

Table 5, panel B, describes the results regarding wealth accumulation across property regimes, thus providing an empirical test of our second set of hypotheses. Column 1 indicates that net wealth increased faster between 2015 and 2018 for couples with a separate rather than community property regime. The gap is no longer statistically significant when we control for the age-related variables (column 2). Therefore, Hypothesis H2a-i is validated: Couples accumulate more wealth with a separate property regime than with a community property regime. However, this is explained by differences in observed characteristics. The difference in wealth accumulation across property regimes remains low (and not statistically significant) after including control variables for labor market outcomes, thus suggesting that Hypothesis H2a-ii is validated: Labor market outcomes explain the wealth accumulation gap across property regimes. Decomposing wealth by asset type yields interesting results, namely that even though we observe no differences in wealth accumulation across property regimes for housing wealth, couples accumulated more financial wealth between 2015 and 2018 with a separate property regime than with a community property regime. This result is in line with Hypothesis H2a-i (for financial wealth), although the difference is not statistically significant. Considering the change in gross wealth instead of net wealth yields similar results (see Table 4 in the online Appendix).

Table 5, panel C, describes the association between the precise marital status (legal status$\times$property regime) and wealth accumulation, thus providing a test for our third set of hypotheses.

Married couples accumulated significantly more wealth over the 2015-2018 period with a separate property regime than with a community property regime. The estimates indicate that PACS and cohabiting couples have also accumulated more wealth than married couples with a community property regime, but this is not statistically significant (column 1). When we control for age-related variables (column 2), the difference between married couples with a community property regime and cohabiting or PACSed couples fades away (and becomes negative). The coefficient remains statistically significant for married couples with a separate property regime, even after controlling for age-related variables (column 2), labor market variables (column 3), and bequest variables (column 4), suggesting that Hypotheses H3a-i and H3b are validated: Married couples with a separate property regime accumulate more wealth than any other type of couple. Controlling for the level of wealth observed in 2015 strengthens the association between marriage with a separate property regime and wealth accumulation (see Table 17 in the online Appendix), which invalidates Hypothesis H3a-ii: Being initially richer does not explain why married couples with a separate property regime accumulate more wealth over the 2015-2018 period.

Regarding other types of couples, we do not observe clear differences: PACS couples with a community property regime accumulate more wealth than married couples with the same regime (the coefficient is large but not statistically significant), which is in line with Hypothesis H3c. Decomposing by asset type indicates that for both housing assets and financial assets, married couples with a separate regime have accumulated more wealth over the 2015–2018 period than married couples with a community property regime, but the estimates are no longer statistically significant. PACS couples with a separate property regime have accumulated less housing wealth than married couples with the same regime, but they have more financial wealth.

Regarding gross wealth, married couples have not accumulated more wealth than other types of couples, as the difference is rather small and not statistically significant (column 2 of Table 7 in the online Appendix). This result suggests that married couples with a separate property regime have less liability than other types of couples. When the amount of debt is controlled for, the difference in gross wealth between married couples with a separate property regime and other couples appears to be slightly larger than when not controlling for debt (column 3).

#### Intrahousehold Wealth Inequality

Table 6 shows the results regarding changes in the female partner’s share of household wealth across couple statuses. Table 4 showed that the female partner’s share of household wealth was similar (and close to 50%) across unmarried cohabitants as well as married and PACSed couples with a community property regime in 2015. For both PACSed and married couples in a separate property regime, the female partner’s share of household wealth was lower than 50%. In Table 6, the first column shows that the gender gap has remained stable between 2015 and 2018 for married couples with a community regime (the estimated constant is equal to 0.001) and for all PACSed couples (with both community and property regimes). The female partner’s share of household wealth increased for married couples with a separate property regime and to a lower extent for cohabiting couples (although the estimates are not statistically significant). Estimates are slightly affected by including characteristics related to age (column 2), labor market (column 3), and bequests (column 4). With the exception of PACSed couples, these results are consistent with Hypothesis H4a: The female partner’s share of household wealth increases for couples in a separate property regime but not for couples in a community property regime. Table 10 in the online Appendix shows that the female partner’s share of household wealth increased for couples with a separate property regime because women accumulated wealth faster than men over the 2015–2018 period.

In order to test Hypotheses H4b-i and H4b-ii, we construct two binary variables. The first one indicates whether the female partner was strictly richer than the male partner in 2015 (i.e., the female partner’s share of the household wealth is larger than 1/2);^17^ the second indicates whether her share of household income is larger than her share of household wealth. Column 5 shows that when the female partner is as rich as (or poorer than) her partner, her share of household wealth remained stable for couples with a community property regime, and it increased for couples in a separate property regime. In couples where the woman is richer than her partner, the female partner’s share of household wealth decreased for all types of couples, and especially so for couples with a separate property regime. This result validates Hypothesis H4b-i: The female partner’s share of household wealth decreases for couples with a community property regime when the women is richer than her partner.

We now compare changes in the female partner’s share of household wealth if her share of household income is larger than her share of household wealth. As expected, the female partner’s share of household wealth has decreased between 2015 and 2018 if her share of household income is lower than her share of household wealth. This is the case for all types of couples, but even more so for couples in a separate property regime. In contrast, the female partner’s share of household wealth increased between 2015 and 2018 in couples where her share of household income was larger than her share of household wealth in 2015, especially for couples with a separate property regime. These results validate Hypothesis H4b-ii: For couples in a separate property regime, the female partner’s share of household wealth increases when her savings capacity (approximated by her share of household income) is larger than her share of household wealth.

We find similar results when studying the female partner’s share of gross household wealth rather than her share of net household wealth (see Table 9 in the online Appendix).

### Additional Analyses

#### Recent Couples

 Our sample is composed of couples where both partners were 25–60 years old in 2015 and combines relationships of different duration. For recently formed couples, the level of wealth observed in 2015 may be close to the initial level of wealth at the time partners met, while couples who formed long before may have accumulated their wealth during the relationship. The people in our sample are either in their first union or have already experienced divorce or separation in the past. In order to better understand how inequality between and within households emerges across couple statuses, we replicated the analysis presented in Tables 5 (panel C) and 6 on a subsample of couples formed within 10 years prior to 2015^18^ who had not experienced a divorce or separation. Notice that in the main analysis, we controlled for the year of couple formation. Restricting our sample to recently formed couples goes beyond controlling for years of couple formation, as it analyzes whether estimates differ for this subsample as compared to the main sample.

The results are presented in Table 11 in the online Appendix. Coefficients are less precisely estimated due to small sample size (220 observations), but results show that married couples accumulated wealth faster with a separate rather than community property regime. Table 12 in the online Appendix partly confirms the results found for the whole sample. Indeed, the female partner’s share of household wealth increased more for both married and PACSed couples with a separate rather than community property regime. As for the analysis on the whole sample, column 5 shows that the female partner’s share of household wealth increased where the women was initially poorer than her partner, but decreased where she was initially richer. Column 6 shows that the female partner’s share increased more with a separate property regime when her share of household income is larger than her share of household wealth.

#### Transition Across Statuses

 In our main analysis, we have focused on couples who did not experience a transition across statuses between 2015 and 2018 and therefore have dropped 125 couples for whom we observe such a transition. This selection affects our results if couples receive a wealth premium when transitioning from one status to another. Several papers have pointed out a positive marriage wealth premium (see for instance Ruel & Hauser (2013) for the USA or Lersch (2017) and Kapelle & Lersch (2020) for Germany.). To investigate this issue, we provide estimates based on a new sample in which we keep the couples who experienced a transition, as long as the couple did not break up during the period. We now focus on the status observed in 2015 and allow the 2018 status to be different. For instance, a couple observed as PACSed with a separate property regime in 2015 but married with a community property regime in 2018 will be classified as a PACSed couple with a separate property regime. We then run the same model on this extended sample. The results (presented in Table 13 in the online Appendix) remain unchanged: Married couples accumulated wealth faster with a separate rather than community property regime, and PACS couples with a separate property regime accumulated financial wealth faster than other couples. Including couples who experienced a transition shows that the potential wealth premium at marriage or PACS is too small to affect our results.

## Concluding Comments

### Discussion of the Results and Limitations

In this study, we analyze how wealth accumulation and the intrahousehold gender wealth gap are related to a couple’s legal status and property regime. This study contributes to the literature by highlighting the relevance of matrimonial property and legal status in shaping economic inequality between and within households. Previous literature has established that marriage is associated with greater wealth than singlehood (see for instance Zagorsky, 2005) and with greater wealth accumulation over time (Kapelle & Lersch, 2020). Cross-country comparisons show that a separate property regime is associated with a larger gender wealth gap within the household when compared with a community property regime (Deere & Doss, 2006).

We use the unique case of France to analyze in a unified framework how both legal status and property regime matter for both wealth accumulation and the gender wealth gap. Using the longitudinal data of the French wealth survey, we followed a sample of couples over the 2015–2018 period in different legal statuses (marriage, civil union, and unmarried cohabitation) and property regimes (community and separate property regimes). Employing multivariate regression analysis, we evaluated how legal statuses and property regimes shape wealth accumulation and the gender wealth gap over the 2015–2018 period in France. This article is the first attempt to compare wealth ownership among three now common marital regimes in France (and elsewhere in Europe): Marriage, non-marital cohabitation and registered partnership (PACS), and property regimes.

Results confirm that married couples hold more wealth than cohabiting couples and bring to light the intermediate position of PACS couples. Results also show that among married and PACS couples, the amount of wealth owned depends on the choice of marital property regime chosen. Married couples with a separate property regime are the wealthiest; unregistered and PACS couples with the same regime are the poorest. Analyzing wealth accumulation over the 2015–2018 period, we show that wealth accumulation exacerbates initial wealth discrepancies: Married couples with a separate property regime accumulated wealth faster than other types of couples, and this is not explained by observed socio-demographic and economic characteristics.

Our results contribute to understanding how the legal status of couples shapes wealth inequality. Different mechanisms are operating simultaneously. For example, cohabiting and PACS couples accumulate wealth faster than married couples, which is in line with the literature arguing that couples accumulate precautionary savings when the risk and cost of separation is higher (González & Özcan, 2013). However, married couples with a separate property regime accumulate more wealth than married couples with a community property regime, as they are wealthier, receive higher returns on their wealth, and have richer families, which is associated with reproduction strategies, including for wealth (Hansen and Toft, 2021). We do not find that cohabiting couples accumulate less housing wealth than married and PACSed couples, suggesting that the choice of official registration is not driven by decisions to invest in housing. If it was the case, we would observe a sudden increase in housing wealth for at least some couples, which would translate into a higher coefficient for PACSed or married than for unregistered cohabitants. All types of couples accumulate housing wealth at a rather similar pace (estimated differences are not statistically significant). Considering housing and financial wealth together, our results are in line with the economic literature studying the impact of unilateral divorce on intrahousehold decision making (Voena, 2015; Stevenson, 2007). In this literature, a decrease in the cost of divorce has a different impact on household savings and investment decisions across property regimes. We show that wealth accumulation differs across property regimes for a given legal status (marriage or PACS), i.e., when the cost of separation induced by the legal status differs across legal statuses.

In the French context, couples can choose from among a variety of couple statuses, which induces some sorting of couples into different statuses. However, differences in wealth accumulation go beyond differences in observed characteristics, and controlling for them does not wipe out differences in wealth accumulation. This suggests that selection into a precise couple status is also based on unobserved characteristics such as risk aversion. It also suggests that both the legal status and property regime create incentives and opportunities to accumulate wealth. It is not clear, however, if the observed differences in our results stem from the unobserved characteristics of couples or from a causal impact of the couple’s status on wealth. Killewald et al. (2017) suggest that the literature needs to establish the causal impact of marriage on wealth accumulation, and we add to this statement by saying that the literature needs to look beyond marriage and establish the causal impact of various couple statuses on wealth accumulation.

In this study, we show that the female partner’s share of household wealth was the lowest among married couples with a separate property regime in 2015. In analyzing how this share changed over the 2015–2018 period, we observe a dynamic toward gender wealth equality within couples: The female partner’s share of household wealth increased in households where she was initially poorer, and it decreased where she was initially richer. The convergence is stronger among couples with a separate property regime (whether unregistered, PACSed, or married) than for couples with a community property regime (married and PACSed).

Our results complement the existing literature on wealth inequality within households by showing its dynamic across couple types. Using cross-sectional data, Frémeaux and Leturcq (2020) showed that between 1998 and 2015, the gender wealth gap remained stable for married and PACSed couples with a community property regime, increased for married and PACSed couples with a separate property regime, and decreased for cohabiting couples. They could not distinguish whether these changes were related to changes in the composition of couples across legal statuses and property regimes or to wealth accumulation during marriage. This current study indicates that the observed increase in wealth inequality within the household is related to the selection of highly unequal couples into marriage with a separate property regime and not to gender discrepancies in wealth accumulation within the household.

The analysis presented in this paper suffers from several limitations. First, the data at hand follows couples over three years, which is rather limited for studying a phenomenon such as wealth accumulation. However, three years were enough to observe some differences between married couples with a separate property regime and other types of couples, suggesting large differences could be found if couples were to be observed for a longer period of time. It hinders the estimation of the dynamics of wealth accumulation across legal statuses and property regimes by separating cohort from period effects. Second, we did not include business assets in our measure of wealth. Most business assets are held by self-employed people, who are more likely to be married with a separate property regime. It is not clear, however, how including business assets would affect our results, as they tend to be more dynamic but riskier. Third, we did not include pension wealth in our measure of wealth. Pension wealth is not observed in the French wealth survey, as it is often the case in wealth surveys. Using German data, Cordova et al. (2022) show that the gender wealth gap is reduced when pension is added. Addind pension wealth should increase the gender wealth gap in France, as the gender pension gap has been found to be substantive at every point in the distribution (Bonnet et al., 2020). Forth, we control for a rather limited number of employment characteristics—employment status and self-employment status of partners. A recent literature has documented the central role of occupations in wealth accumulation (Waitkus & Minkus, 2021). As occupation classes may also be related to the choice of couple type, it raises the question on how occupation classes, couple status and wealth accumulation are intertwined.

### Generalization of the French Case

Why does France offer a unique case to study in a unified framework the impact of legal status and property regimes on wealth accumulation and the gender wealth gap? First, France offers a unique context with a variety of legal statuses and property regimes for couples, who routinely choose the legal framework that best suits their union. France differs from other European countries in the number of legal options offered to opposite-sex couples, with several statuses being made available (marriage and PACS), and unregistered cohabitation is largely accepted. Exploring 23 legislations in Europe as of 2015^19^, Waaldijk (2017) indicates that only four of them had created a registered partnership for opposite-sex couples similar to the French PACS, namely Belgium (2000), Greece (2008), Malta (2014) and the Netherlands (1998). Many other European countries have created a registered partnership, but for same-sex couples only. In countries where civil unions are available for opposite-sex couples, they are unequally popular. In France, 46% in 2018 of opposite-sex couples establishing an official relationship opted for civil unions. This was 48% in 2019 in Belgium, a country where civil unions are as popular as in France,^20^ and 19% in 2016 in the Netherlands.^21^ Civil unions across European countries share some common features, most notably that they are typically less protective than marriage and are easier to dissolve.

The laws regulating unregistered or registered cohabitation and marriage differ across countries. Comparing nine European jurisdictions^22^ and four policy areas^23^, Perelli-Harris & Gassen (2012) explore the quantity and coherence of policies regulating unregistered or registered cohabitation and compare them to marriage. Their results show that the French PACS is one of the most regulated type of union in the nine European jurisdiction under scrutiny. It is not as regulated as registered cohabitation in the Netherlands, but it is similar to unregistered cohabitation in Norway and Sweden. On the contrary, unregistered cohabitation in France is one of least regulated types of cohabitation. It is similar to unregistered cohabitation in Switzerland and Germany. We expect our theoretical framework to be generalizable to countries where civil unions are available to opposite-sex couples, and partly to countries where only marriage and unregistered cohabitation are available to couples.

Additionally, PACSed and married couples in France can choose between a community property regime (the default option for married couples) or separate property regime (the default for PACSed couples). A prenuptial agreement is required to opt out of the default property regime, and this comes at a moderate cost in France. Signing a prenuptial contract is becoming increasingly common in France, with roughly 20% of French newlywed couples having opted out of the default property regime in the 2010s (Frémeaux & Leturcq, 2018). Little is known regarding the prevalence of prenuptial agreements in other European countries-with the notable exception of Italy, where it has been well-documented that newlywed couples massively opt out of the default property regime (community of acquisitions) and choose to separate their assets (Fraboni and Vitali, 2019; Bayot & Voena, 2015), especially couples who transition to marriage after experiencing a period of non-marital cohabitation (Vitali & Fraboni, 2022). France and Italy are comparable because community of acquisitions is the default property regime, and couples opting out of the default regime in both countries share similar traits: They are more educated, more likely to be self-employed, and more likely to be both active in the labor market. We expect our theoretical framework to be generalizable to European countries with a default community of acquisitions regime and the couples who opt out of the default choose a separate property regime. It remains unclear whether it would be generalizable to European countries with another type of default property regime, such as community of accrued gains. In Germany, where the default marital contract is the community of accrued gains, Nutz et al. (2022) found that 5% of all married couples opt out of the default marital contract. Approximately half of them opt for a separate property regime, which is lower than in France or Italy. More research is needed to understand the prevalence and the determinants of opting out of the default property regimes across countries, in relation with the type of default marital contract.

Second, France provides a unique case to study in a unified framework the impact of legal status and property regimes on wealth accumulation and the gender wealth gap because it offers a unique data source for studying wealth, due mainly to three main features: a) the data is longitudinal; b) it allows measuring wealth at the individual level; and 3) it provides precise information on the legal statuses and property regimes of couples. These features allow reconstructing personal wealth from a list of assets held by the household. Measuring personal wealth at the asset level provides more precise estimates of personal wealth than general questions on how aggregate wealth is distributed between spouses. The French wealth survey provides a nice example of how incorporating a few questions on how assets are shared (as suggested in Doss et al., 2011) yields interesting and tractable information at the individual level. Yet, the French wealth survey does not distinguish between legal ownership and actually being able to use and control wealth. Community property regimes protect women from being disadvantaged by compensating for their having a lower savings capacity than men. However, separate property regimes grant women the right to manage their own assets. The wealth survey could be improved by introducing additional questions related to wealth management within the household.

To conclude, our study calls for a better examination of the role that property regimes play in marital wealth accumulation, and it suggests that an increased diversity of marital statuses over the life course may have important consequences on wealth inequality between individuals. Diversification in marital statuses is not specific to France, as cohabitation rates have been increasing in the developed world due to the creation of civil unions in many European and non-European countries. Furthermore, an increasing share of couples are signing prenuptial agreements in other countries. The data at hand are too limited to study wealth premiums and the dynamics of wealth accumulation over time across couple statuses, but future research can explore these promising topics.

**Table 1:** The legal features of marital statuses in France

	Cohabitation	PACS	Marriage
Income taxation	Separate	Joint, starting from the year the PACS is registered	Joint, starting from the year the marriage is registered
Inheritance	$\bullet$ Surviving partner must be declared in the will $\bullet$ High tax rates	$\bullet$ Surviving partner must be declared in the will $\bullet$ Low tax rates, same as marriage	$\bullet$ Surviving partner automatically inherits from the spouse $\bullet$ Low tax rates, same as PACS
Asset sharing	No asset sharing, unless bought together	$\bullet$ Before 2007: default community property regime $\bullet$ Since 2007: default separate property regime $\bullet$ Can be changed when contracting the PACS	$\bullet$ Default community property regime $\bullet$ Couples can sign a prenuptial agreement and opt for either a separate or full community regime
Debts	No solidarity	Solidarity of debts linked to everyday life and housing	Solidarity of debts (but protection for housing)
Adoption of children	No legal adoption by partners (but one can adopt independently)	No legal adoption by partners (but one can adopt independently)	Legal adoption
Social protection	No common coverage	Common coverage	Common coverage
Survivor’s pension	No	No	Yes
Citizenship	No citizenship	No citizenship, but being PACSed can be relevant	Citizenship after 4 years
Dissolution	Unilateral or mutual. No cost, but no alimony or other compensation	Unilateral or mutual. Low cost: letter to the court. But no financial compensation, possibility of damages pension	Mutual. Divorce costs. Ex- spouses can claim compensatory benefits.
Miscellaneous		Created in 1999	Same-sex marriage allowed in 2013

*Legal features at the end of 2015. From Laws and Families Database*https://lawsandfamilies-database.site.ined.fr/en/legal-project/interactive-database/

**Table 2:** Hypotheses on the associations between a couple’s legal status and their property regime, wealth accumulation, and the gender wealth gap

	Cohabitation	PACS		Marriage	
*Wealth accumulation by couple legal status*					
H1a-i	++ $^{(1)}$	+		+	
H1a-ii		++ $^{(1)}$		+	
H1b	+	++ $^{(1)}$		++ $^{(1)}$	

	Separate			Community	
*Wealth accumulation by couple property regime*					
H2a-i	++ $^{(2)}$			+	
H2a-ii	+ $^{(3)}$			+	
H2b	+			++ $^{(3)}$	

	Cohabitation	PACS		Marriage	
		Separate	Community	Separate	Community
*Wealth accumulation by couple legal status and property regime*					
H3a-i	+	+	+	++	+
H3a-ii	+	+	+	+ $^{(4)}$	+
H3b	+	+		++	
H3c			++		+

	Separate			Community	
*Gender wealth gap by couple legal status and property regime*					
H4a	++			+	
H4b-i	++ $^{(5)}$				
H4b-ii				++ $^{(6)}$	

$^{(1)}$ driven by higher financial wealth; $^{(2)}$ driven by higher income; $^{(3)}$ controlling for household income ; $^{(4)}$ controlling for level of wealth ; $^{(5)}$ when the female partner’s share of household income is larger than the female partner’s share of wealth in 2015; $^{(6)}$ when the female partner’s share of household wealth is lower than 1/2 in 2015

**Table 3:** Descriptive statistics

		Unmarried	Married	Married	Pacs	Pacs
		cohab.	comm.	sep.	comm.	sep.
Household income ($\times$ 1,000):						
2015		34	44	61	54	48
2018		37	48	69	59	54
Man’s age		40.7	46.4	45.7	39.6	36.1
Woman’s age		39.1	44.3	42.8	38.5	34.8
Year of couple formation:						
bef. 1979		.001	.108	.013	0	0
1980–1989		.082	.291	.207	.118	.033
1990–1999		.202	.317	.338	.3	.116
2000–2009		.416	.241	.396	.446	.578
2010 or after		.299	.042	.045	.137	.274
Past divorce		.278	.11	.252	.14	.187
Man’s education:						
Low		.587	.543	.227	.233	.356
Medium		.177	.185	.21	.302	.232
High		.236	.272	.562	.466	.412
Woman’s education:						
Low		.578	.507	.299	.103	.273
Medium		.147	.186	.163	.257	.162
High		.275	.308	.538	.64	.565
Currently employed (in 2015):						
Man		.817	.826	.932	.951	.937
Woman		.728	.744	.844	1	.911
Self-employment (in 2015):						
Man		.209	.169	.44	.094	.106
Woman		.053	.069	.224	.099	.052
Number of children in the household (in 2015):						
0		.291	.269	.185	.045	.307
1		.363	.227	.267	.25	.306
2		.23	.37	.415	.677	.34
3 or more		.116	.134	.133	.028	.042
Has received bequest during 2015–2018		.079	.070	.079	0	.059
Has made donation during 2015–2018		0	.009	.017	.079	0
N		204	1102	221	31	108
Share (weighted)		16.7%	65.8%	7.9%	1.9%	7.6%

Data: *Patrimoine* surveys 2014–2015 and 2017–2018. Notes: The sample is restricted to couple-headed households with no change in status between 2015 and 2018, and both partners were between 25 and 60 years old in 2015

**Table 4:** Descriptive statistics on household net wealth and female partner’s share of household net wealth

		Unmarried	Married	Married	Pacs	PACS
		cohab.	comm.	sep.	comm.	sep.
Net household wealth ($\times$ 1,000):						
2015	Mean	140	206	373	191	142
	SD	(249)	(250)	(512)	(288)	(270)
2018	Mean	174	227	449	220	164
	SD	(226)	(232)	(608)	(248)	(268)
Change 2015–2018		24.3%	10.2%	20.4%	15.2%	15.5%
Net household housing wealth ($\times$ 1,000):						
2015	Mean	104	166	287	138	105
2018	Mean	137	186	327	168	121
Change 2015–2018		31.7%	12%	13.9%	21.7%	15.2%
Net household financial wealth ($\times$ 1,000):						
2015	Mean	35.4	40.6	85.6	53.4	37.1
2018	Mean	37.2	41.1	122	52	43.3
Change 2015–2018		5%	1.2%	42.5%	-2.6%	16.7%
Female partner’s wealth share:						
2015		.477	.483	.414	.507	.423
2018		.509	.484	.439	.508	.424
Change 2015–2018		.032	.001	.025	.001	.001

Data: *Patrimoine* surveys 2014-2015 and 2017-2018. The sample is restricted to couple-headed households with no change in status between 2015 and 2018, and both partners were between 25 and 60 years old in 2015

**Table 5:** Couple types and wealth accumulation (2015-2018)

	(1)	(2)	(3)	(4)	(5)	(6)
	Net	Net	Net	Net	Net housing	Net financial
	wealth	wealth	wealth	wealth	wealth	wealth
*Panel A: Legal status of couples*						
Unregist. cohab.	0.102	−0.088	−0.121	−0.128	0.079	0.088
	(0.101)	(0.112)	(0.115)	(0.115)	(0.117)	(0.160)
PACS	0.135	−0.110	−0.102	−0.083	−0.025	0.388**
	(0.128)	(0.138)	(0.139)	(0.140)	(0.142)	(0.194)
Married	*Ref.*	*Ref.*	*Ref.*	*Ref.*	*Ref.*	*Ref.*
*Panel B: Property regime of couples*						
Comm. property	*Ref.*	*Ref.*	*Ref.*	*Ref.*	*Ref.*	*Ref.*
Sep. property	0.210***	0.072	0.028	0.028	0.056	0.186
	(0.080)	(0.090)	(0.091)	(0.092)	(0.093)	(0.127)
*Panel C: Legal status* $\times$ *Property regime*						
Unregist. cohab.	0.144	−0.039	−0.083	−0.090	0.092	0.112
	(0.102)	(0.114)	(0.116)	(0.117)	(0.119)	(0.162)
Married (comm. assets)	*Ref.*	*Ref.*	*Ref.*	*Ref.*	*Ref.*	*Ref.*
Married (sep. assets)	0.399***	0.357**	0.307**	0.320**	0.163	0.151
	(0.140)	(0.142)	(0.148)	(0.148)	(0.150)	(0.205)
PACS (comm. assets)	0.168	0.014	0.051	0.130	0.351	0.259
	(0.270)	(0.272)	(0.273)	(0.278)	(0.282)	(0.385)
PACS (sep. assets)	0.179	−0.081	−0.087	−0.080	−0.096	0.452**
	(0.142)	(0.154)	(0.155)	(0.155)	(0.158)	(0.215)
Demographics		X	X	X	X	X
Professional			X	X	X	X
Bequest				X	X	X
Observations	1666	1666	1666	1666	1666	1666
$R^{2}$	0.001	0.029	0.041	0.048	0.087	0.073

Data: *Patrimoine* surveys 2014–2015 and 2017–2018. Note: Standard errors in parentheses. * p<.1, ** p<.05, *** p<.01. Demographic characteristics include: male partner’s birth cohort, age difference between partners, year of couple formation, size of residence city, citizenship of both partners, and past marriage. Professional characteristics include: educational attainment of both partners, 2015 household income (in log), changes in household income (2015–2018), at least one partner is self-employed in 2015, change in self-employment (2015–2018), at least one partner is not working full-time in 2015, and change in full-time employment (2015–2018). Bequest characteristics include: dummy variable for receiving a bequest or a gift (2015–2018), dummy variable for giving an inter-vivos gift (2015–2018), number of children in the household in 2015, binary for a decrease in the number of children (2015–2018), and binary for an increase in number of children (2015-2018). Wealth is expressed in 1,000 euros (2015), IHS-transformed, and constructed as follows. Net wealth: the sum of all the couple’s assets minus liabilities (excluding business assets). Net housing wealth: the sum of all the couple’s housing assets minus housing debt. Net financial wealth: the sum of all the couple’s financial assets minus non-housing debt

**Table 6:** Changes in female partner’s share of household wealth across couple’s statuses (2015–2018)

	(1)	(2)	(3)	(4)	(5)	(6)
	Female	Female	Female	Female	Female	Female
	share	share	share	share	share	share
Cohab.	0.011	0.011	0.012	0.015	0.090***	−0.038**
	(0.012)	(0.013)	(0.014)	(0.014)	(0.015)	(0.016)
Married (comm.)	*Ref.*	*Ref.*	*Ref.*	*Ref.*	*Ref.*	*Ref.*
Married (sep.)	0.023	0.027*	0.023	0.023	0.062***	−0.042*
	(0.016)	(0.016)	(0.017)	(0.017)	(0.018)	(0.022)
PACS (comm.)	−0.000	0.000	−0.002	−0.007	−0.016	0.005
	(0.030)	(0.031)	(0.031)	(0.031)	(0.031)	(0.038)
PACS (sep.)	0.001	−0.006	−0.004	−0.003	0.059***	−0.066***
	(0.016)	(0.018)	(0.018)	(0.018)	(0.019)	(0.021)
F richer than M					−0.125***	
					(0.022)	
Cohab. $\times$ F richer than M					−0.096***	
					(0.030)	
Married (sep.) $\times$ F richer than M					−0.093**	
					(0.041)	
PACS (comm.) $\times$ F richer than M					0.101	
					(0.090)	
PACS (sep.) $\times$ F richer than M					−0.126***	
					(0.039)	
F inc. sh. > F W. sh.						0.054***
						(0.011)
Cohab. $\times$ F inc. sh. > F W. sh.						0.136***
						(0.024)
Married (sep.) $\times$ F inc. sh. > F W. sh.						0.112***
						(0.031)
PACS (comm.) $\times$ F inc. sh. > F W. sh.						−0.038
						(0.060)
PACS (sep.) $\times$ F inc. sh. > F W. sh.						0.159***
						(0.033)
Constant	0.001	−0.002	0.019	0.013	0.008	−0.057
	(0.005)	(0.022)	(0.052)	(0.053)	(0.049)	(0.051)
Demographics		X	X	X	X	X
Professional			X	X	X	X
Bequest				X	X	X
Observations	1612	1612	1612	1612	1612	1612
$R^{2}$	0.002	0.016	0.032	0.037	0.166	0.135

Data: *Patrimoine* surveys 2014–2015 and 2017–2018. Note: Standard errors in parentheses. * p<.1, ** p<.05, *** p<.01. Demographic characteristics include: male partner’s birth cohort, age difference between partners, year of couple formation, size of residence city, citizenship of both partners, and past marriage. Professional characteristics include: educational attainment of both partners, 2015 household income (in log), changes in household income (2015–2018), at least one partner is self-employed in 2015, change in self-employment (2015–2018), at least one partner is not working full-time in 2015, and change in full-time employment (2015–2018). Bequest characteristics include: dummy variable for receiving a bequest or a gift (2015–2018), dummy variable for giving an inter-vivos gift (2015–2018), number of children in the household in 2015, binary for a decrease in the number of children (2015–2018), and binary for an increase in number of children (2015-2018). Female partner’s share is her share of the net household wealth. Net wealth is constructed as the sum of all the couple’s assets minus liabilities (excluding business assets)

### Supplementary Information

Below is the link to the electronic supplementary material.

Supplementary file1 (PDF 267kb)

## A Appendix

### A.1 Wealth Accumulation

Let $W_{it}$ represent wealth of couple *i* at beginning of period *t*. $W_{it+1}$ is wealth of couple *i* at beginning of period $t+1$.

$W_{it+1}$ can be written as:

$$\begin{aligned} W_{it+1} = (1+r_{it})W_{it} + S_{it} + B_{it} \end{aligned}$$

where $S_{it}$ gives savings on earnings during period *t* and $B_{it}$ represents bequests or donation received or given by the couple *i* during period *t*. $S_{it}$ and $B_{it}$ can be positive or negative. $r_{it}$ is a composite rate of return for couple *i* during period *t*. It depends on the wealth portfolio of couple *i*. Couple *i* owns $W_{iat}$ of asset *a* at period *t* and $r_{at}$ is the return of asset type *a* during period *t*. The composite rate of return $r_{it}$ can be written as: $r_{it} = \sum _a r_{at} \times \frac{W_{iat}}{W_{it}}$. The composite rate of return depends on the return of each type of assets, and the wealth portfolio $\{\frac{W_{iat}}{W_{it}}\}_{a \in A}$ of couple *i*.

Household savings on earnings are composed of savings on female partner’s earnings and savings on male partner’s earnings. $S_{it}$ can be written as $S_{it} = S_{it}^f + S_{it}^m$, with $S_{it}^f$ (resp. $S_{it}^m$) is savings on female partner’s (resp. male partner’s) earnings in couple *i*. Similarly, wealth transfers received or given by the household are composed of wealth transfers received or given by the female partner and wealth transfers received or given by the male partner. $B_{it} = B_{it}^f + B_{it}^m$, with $B_{it}^f$ (resp. $B_{it}^m$) is female partner’s (resp. male partner’s) wealth transfer received or given.

Now let’s define wealth accumulation at the individual level. $W^j_{it}$ represents wealth of partner *j* ($j \in \{f,m\}$) at beginning of period *t*. We define $\beta ^j_{it}$ as the share of the couple *i*’s savings that goes to individual *j*. If the couple is married with a community regime, all acquired assets are jointly held which means they share equally all savings and $\beta ^j_{it} = 1/2$ for $j\in \{f,m\}$. If the couple is cohabiting, or married (or PACSed) with a separate assets, all acquired assets remains a personal asset, which means that $\beta ^j_{it} = S_{it}^j/S_{it}$.

Wealth $W^j_{it+1}$ can be written as:

$$\begin{aligned} W^j_{it+1} = (1+r^j_{it})W^j_{it} + \beta ^j_{it} S_{it} + B^j_{it} \end{aligned}$$

Notice that $W^j_{it}$ is composed of joint and personal assets. As the wealth portfolio may differ across partners, $r^j_{it}$ may also differ across members within the household.

### A.2 Woman’s Share of Household Wealth

Let’s $\alpha _{it}$ represent the woman’s share of household wealth at the beginning of period *t*. It is defined as:

$$\alpha _{it} = \frac{W_{it}^f}{W_{it}}$$

In period $t+1$, the female partner’s share of the household wealth has become:

2 $$\begin{aligned}{\alpha _{it + 1}} &= \frac{{W_{it + 1}^f}}{{{W_{it + 1}}}}\\ &= \frac{{(1 + r_{it}^f)W_{it}^f + \beta _{it}^f{S_{it}} + B_{it}^f}}{{(1 + {r_{it}}){W_{it}} + {S_{it}} + {B_{it}}}}\\ & = \underbrace {\frac{{W_{it}^f}}{{{W_{it}}}}}_{ = {\alpha _{it}}}\underbrace {\frac{{(1 + r_{it}^f) + \frac{{\beta _{it}^f{S_{it}} + B_{it}^f}}{{{\alpha _t}{W_{it}}}}}}{{(1 + {r_{it}}) + \frac{{{S_{it}} + {B_{it}}}}{{{W_{it}}}}}}}_{ \equiv {K_{it}}} \end{aligned}$$

The female partner’s share of the household’s wealth increases if $K_{it} > 1$, which can be rewritten as:

$$\begin{aligned} K_{it}> 1 \Leftrightarrow (r^f_{it}-r_{it})\alpha _{it} W_{it} + (\beta ^f_{it}-\alpha _{it})S_{it} + (B^f_{it}-\alpha _{it} B_{it}) > 0 \end{aligned}$$

This inequality shows that the female partner’s share of the household wealth changes under the combined effect of three elements:

1. $(r^f_{it}-r_{it})\alpha _{it} W_{it}$: this element is positive if $r^f_{it}-r_{it}>0$, i.e., if the female partner’s wealth is more dynamic than household’s wealth.
2. $(\beta ^f_{it}-\alpha _{it})S_{it}$: this element is positive if $S_{it}>0$ and $\beta ^f_{it}-\alpha _{it}>0$, i.e., the female partner’s share of savings is larger than the female partner’s share of wealth. If the couple is married or PACSed with a community property regime, $\beta ^f_{it}=1/2$: equal sharing of savings tends to increase the female partner’s share of household wealth if the female partner is initially poorer than the male partner (i.e., if $\alpha _{it}<1/2$). If the couple is cohabiting or married or PACSed with a separate property regime, $(\beta ^f_{it}-\alpha _{it})$ is positive if her share of household earnings is larger than her share of household wealth. To state it in a simple way, if the female partner holds 30% of initial wealth and 40% of household’s earnings, then her share of household’s wealth increases.
3. $(B^f_{it}-\alpha _{it} B_{it})$: the same reasoning applies here. If $B_{it}$ is positive (the household received wealth transfers) and if the female partner’s share of wealth transfers is larger than her share of initial wealth, then $(B^f_{it}-\alpha _{it} B_{it})$ is positive.

## Footnotes

1 The remainder of this paper will use the terms “unmarried cohabitation”, “unmarried couple”, “unregistered cohabiting couple”, and “cohabiting couple” in reference to couples who are neither married nor PACSed.

2 PACS stands for *Pacte Civil de Solidarité* and is colloquially referred to as “getting PACSed”. It was introduced in 1999 as a legal alternative to unmarried cohabitation and marriage. Its main goal was initially to provide some legal recognition to same-sex couples without legalizing same-sex marriage (which did not occur in France until 2013), and was also made available to opposite-sex couples. In 2018, 96% of PACS were opposite-sex couples (Papon & Beaumel, 2020). It is worth noting that PACS did not exist when the older couples in our sample married each other, but some of them would have chosen it. Throughout the text, couples who have signed a PACS will be referred to as “PACSed couples”.

3 For the sake of simplicity, community property regime encompasses two types of community regimes: community of acquisitions and full community (in which all assets are jointly owned by the husband and wife, including bequests, gifts, and assets acquired before marriage). Here, we combine the two regimes because only less than 2% of all married couples opt for a full community property regime (Frémeaux & Leturcq, 2013). Thus, we will use the terms “community of acquisitions regime” and “community property regime” interchangeably.

4 Let $r_{at}$ be the return on the asset of type *a* in period *t*. Couple *i* owns $W_{iat}$ of asset *a* at period *t*. The composite rate of return $r_{it}$ can be written as: $r_{it} = \sum _a r_{at} \times \frac{W_{iat}}{W_{it}}$. The composite rate of return depends on the return on each type of asset and on the wealth portfolio $\{\frac{W_{iat}}{W_{it}}\}_a$ of couple *i*.

5 In France, marriage remains the most common context for raising children, but starting a family out of marriage has become standard over recent decades (Bodier et al., 2015). In 2020, 62.2% of new births occurred out of wedlock (see https://www.insee.fr/fr/statistiques/2381394). However, the share of couples who marry after a child is born has been increasing over time: 28% of newly wed couples over the 2004-2013 period already had a child, while this was only 1% for marriages celebrated in 1964–1973 (Maillochon, 2019).

6 The most dynamic assets are equities, shares, and bonds, with an average annual rate of return of 9.4%. However, Garbinti et al. (2021) show that only the richest 1% of the population hold large amounts of these asset types, while the vast majority of the population store their wealth in deposits, life insurance, and housing assets.

7 Let us illustrate this point with a numerical example. Imagine a couple starting a relationship with personal wealth of 9 for the male partner and 1 for the female partner. The female partner’s share of household wealth is 1/(9+1) = 10% at couple formation. If they accumulate joint wealth of value 100 over time, her share of household wealth becomes (50+1)/(100+9+1) = 46%.

8 The French wealth survey is a cross-sectional household survey that has collected data every six years since 1986, with seven waves currently available: 1986, 1992, 1998, 2004, 2010, 2014-2015, and 2017-2018. From 2014 onward, the data are combined into cross-sectional and longitudinal components. Each wave includes roughly 12,000 households, with half of them being followed up over time (roughly 6,000). For the purpose of this study, we use only the longitudinal component.

9 Excluding couples transitioning across statuses means that we do not use these transitions to estimate premiums for being in a specific legal status, as in Lersch (2017). Although we follow previous studies evaluating how marriage stratifies wealth by comparing married to unmarried couples, we consider a greater variety of couple statuses. In Sect. 5.3, we run additional analyses that include couples experiencing marital transitions in our sample.

10 Few values for wealth-related variables are missing because INSEE (the data producer of the French wealth survey) provides imputations in case of missing values, asset by asset. The survey is designed to study wealth, which means that it did not include households unwilling to answer wealth-related questions. More information regarding imputation is provided in online Appendix.

11 There is only one respondent per household (either the man, the woman, or the couple together), so we do not observe inconsistencies in values reported by two different respondents. However, self-reported values lead to underestimates of both aggregate wealth and the level of wealth inequality. For France, the European Central Bank (2013) estimates that the mean net wealth per capita is 1/3 lower in the French wealth survey than in national accounts. The richer the household, the larger the share of missing wealth in the survey. Using the French wealth survey, Frémeaux & Leturcq (2020) find a top 10% wealth share of 45% in 2015, Garbinti et al. (2021) constructed distributional national accounts to estimate a top 10% wealth share of 55%.

12 Business assets are excluded because the individualization of these assets requires supplementary assumptions. Because individualizing wealth is required for analyzing the gender wealth gap, we exclude business assets for the sake of consistency in our two analyses (wealth accumulation and gender gap).

13 Before performing the IHS transformation, Aihounton & Henningsen (2020) suggest using the unit of measurement that gives the best fit of the model. We regressed wealth expressed in different units (euros, hundreds of euros, thousands of euros, and tens of thousands of euros) before IHS transformation on dummies for marital status and property regimes. We selected wealth expressed in thousands of euros because it gave the highest $R^2$.

14 Educational attainment and citizenship are not fixed over time, but they do not vary for most observations in our sample.

15 Marriage with a separate property regime is a convenient way to protect household wealth in case of professional bankruptcy. The association between self-employment and separate property regime is historically long-standing, as already noted by Barthez & Laferrère (1996) and Frémeaux & Leturcq (2013).

16 Recall $IHS(x) = ln(x+\sqrt{x^2 +1})$ and let ${\tilde{x}} = (1+g)x$. We derive: $IHS({\tilde{x}})-IHS(x) \approx ln(\frac{{\tilde{x}}}{x})$ for large and positive ${\tilde{x}}$ and *x*, and $ln(\frac{{\tilde{x}}}{x}) \approx g$ for small *g*. In other words, the left-hand size variable can be interpreted as the growth rate of wealth. The coefficients $\beta _k$ indicate how the growth rate of wealth (denoted *g*) differ across couple’s types.

17 We constructed the binary variable to be equal to one when the female partner’s share of household wealth is initially larger than 52.5%. Therefore, the variable compares women who are at least 10% richer than their partners, relative to women who are as or less wealthy than their partners.

18 We consider the date of couple formation. That is, for couples cohabiting before marriage, we consider the date when their cohabitation started.

19 Austria, Belgium, Bulgaria, Czech Republic, Finland, France, Germany, Greece, Hungary, Iceland, Ireland, Italy, Malta, Netherlands, Norway, Poland, Portugal, Romania, Slovenia, Sweden, UK: England and Wales, UK: Northern Ireland, UK: Scotland. See: https://lawsandfamilies-database.site.ined.fr/en/

20 https://statbel.fgov.be/fr/nouvelles/moins-de-mariages-plus-de-cohabitations-legales-en-2019, consulted on Dec 17, 2021.

21 https://www.cbs.nl/en-gb/news/2017/34/registered-partnership-gaining-popularity, consulted on Dec 17, 2021.

22 Austria, England, France (registered and unregistered cohabitation), Germany, Netherlands (registered and unregistered cohabitation), Norway, Spain, Sweden, Switzerland

23 Rights and responsibilities during the union, Rights and responsibilities after a union dissolves, Laws regulating what happens after the death of a partner and, Laws regulating the relationship between parents and children.
