# Supplementary material for: Wealth Accumulation and the Gender Wealth Gap Across Couples’ Legal Statuses and Matrimonial Property Regimes in France
Source: Eur J Popul. 2022 Aug 9;38(4):643–79. doi: 10.1007/s10680-022-09632-5 (PMC9550916; doi:10.1007/s10680-022-09632-5)
Supplement: Supplementary file 1 — Supplementary file1 (PDF 267kb) [file 10680_2022_9632_MOESM1_ESM.pdf]

B Online Appendix

B.1 Imputation of missing values in the French Wealth Survey

The imputation of missing values in the French Wealth survey is done by the Insee, producer of the survey. The French Wealth Survey addresses several issues of non-response. Regarding total non-response, the success rate of the French Wealth survey is 67.2% in 2014-2015: 12,084 households answered the survey over the 17,987 households included in the survey. Approximately 50 households refused to declare any asset or debt—these households are excluded from the survey and considered total non-response. Total non-response is corrected using survey weights.

The survey distinguishes two types of partial non-response: households not declaring some assets and debts, and not declaring the value of their assets. The issue of households not declaring some assets is corrected using hot-deck method. This type of correction is rather rare and we do not consider these imputed assets.

Regarding the value of assets, questions depend on the type of asset. Regarding real-estate assets, the household was invited to give the value into brackets. It could provide an exact value by giving the same value for the min and max of the bracket. Regarding financial assets, households were first invited to give the exact value of the asset. If they did not know or did not want to give the exact value of the asset, it could provide a value into brackets. For both type of assets, when the value is given into brackets, the exact value is then imputed using a simulated residual method, and a truncated normal distribution to simulate the residual. When the household did not provide any information on the value of asset, the value of the asset was estimated using information on the value of assets with similar characteristics observed for similar households. Table B.1 indicates, for each type of asset, the share of assets for which the exact value or a value into bracket was provided by the household, and when the value had to be imputed. Overall, the producer had to impute the value of a real-estate asset in less than 20% of cases, and less than 10% of cases for financial assets. The share

Table B.1: Imputation of housing and financial assets and debts in the French Wealth Survey (2015–2018)

Type of asset or debt	Self-declared exact value	Self-declared value by bracket	No self-declared value		N
			Ex-post estimation	Unknown	
**2014-2015**					
Real-estate assets	18.8%	63.5%	17.7%		16,243
Mean value	*314,022*	*288,081*	*291,716*		
Financial assets	75.3%	15.0%	8.2%	1.5%	89,443
Mean value	*33,006*	*30,137*	*78,401*		
Housing debt	95.1%		<0.1%	4.8%	7,360
Mean value	*120,924*				
Financial debt	81.6%			18.3%	4,005
Mean value	*19,009*				
**2017-2018**					
Real-estate assets	27.9%	56.6%	15.4%		19,025
Mean value	*297,565*	*302,948*	*314,366*		
Financial assets	75.9%	13.9%	8.9%	2.2%	104,743
Mean value	*37,075*	*62,510*	*112,266*		
Housing debt	94.9%			5.1%	8,112
Mean value	*133,437*				
Financial debt	94.7%			5.3%	4,431
Mean value	*17,307*				

of assets for which information was not sufficient to impute a value is rather rare and represents less than 5% of cases. Regarding household debts, the value had to be imputed for approximately 5% of cases for housing debts and 18% of cases for financial debts.

Table B.2: Legal status and net wealth accumulation (full table)

	(1) Net wealth		(2) Net wealth		(3) Net housing wealth		(4) Net financial wealth	
Unregist. cohab.	0.10	(0.10)	-0.13	(0.12)	0.08	(0.12)	0.09	(0.16)
PACS	0.13	(0.13)	-0.08	(0.14)	-0.02	(0.14)	0.39**	(0.19)
Married	*Ref.*		*Ref.*		*Ref.*		*Ref.*	
Birth cohort (man): 1950–1959			-0.13	(0.14)	-0.03	(0.14)	-0.19	(0.20)
Birth cohort (man): 1960–1969			*Ref.*		*Ref.*		*Ref.*	
Birth cohort (man): 1970–1979			0.26**	(0.12)	0.34***	(0.12)	-0.01	(0.16)
Birth cohort (man): 1980 or aft.			0.38**	(0.16)	0.97***	(0.16)	-0.40*	(0.23)
Age diff: M-F≥10			-0.24	(0.20)	0.19	(0.21)	-0.07	(0.28)
Age diff: M-F∈[3,10[			-0.08	(0.09)	0.07	(0.09)	-0.01	(0.12)
Age diff: M-F∈[-2,3[			*Ref.*		*Ref.*		*Ref.*	
Age diff: M-F<-2			-0.01	(0.13)	-0.07	(0.13)	0.10	(0.18)
Couple form.: bef. 1980			*Ref.*		*Ref.*		*Ref.*	
Couple form.: 1980–1989			0.23	(0.18)	0.21	(0.19)	0.04	(0.26)
Couple form.: 1990–1999			0.20	(0.21)	0.15	(0.21)	-0.40	(0.29)
Couple form.: 2000–2009			0.45**	(0.23)	0.37	(0.23)	-0.34	(0.32)
Couple form.: 2010 or aft.			0.62**	(0.26)	0.22	(0.27)	-0.13	(0.36)
Rural			*Ref.*		*Ref.*		*Ref.*	
Less than 20,000			-0.05	(0.11)	0.03	(0.11)	0.18	(0.15)
20,000-100,000			-0.07	(0.13)	-0.19	(0.13)	0.29	(0.18)
More than 100,000 (excl. Paris)			0.10	(0.10)	0.04	(0.11)	0.17	(0.14)
Paris and suburbs			0.15	(0.13)	0.31**	(0.13)	-0.14	(0.18)
Ever married			0.12	(0.12)	0.10	(0.12)	-0.17	(0.16)
Edu. (man): Low (ISCED 1–2)			*Ref.*		*Ref.*		*Ref.*	
Edu. (man): Medium (ISCED 3–4)			-0.22**	(0.11)	-0.33***	(0.11)	0.29**	(0.15)
Edu. (man): High (ISCED 5–8)			-0.15	(0.10)	-0.10	(0.10)	-0.14	(0.13)
Edu. (woman): Low (ISCED 1–2)			*Ref.*		*Ref.*		*Ref.*	
Edu. (woman): Medium (ISCED 3–4)			-0.03	(0.11)	0.05	(0.11)	-0.14	(0.15)
Edu. (woman): High (ISCED 5–8)			-0.05	(0.10)	0.07	(0.10)	0.02	(0.14)
Chg in hh income (2018–2015)			0.01	(0.03)	0.06**	(0.03)	0.00	(0.04)
Log of hh income (2015)			0.08**	(0.04)	0.09**	(0.04)	0.07	(0.05)
Self-emp. in 2015			0.26***	(0.09)	0.41***	(0.10)	-0.08	(0.13)
Self-emp. in 2015, not in 2018			-0.53*	(0.32)	-0.68**	(0.32)	-0.19	(0.44)
Not self-emp. in 2015, self-emp. in 2018			-0.10	(0.22)	-0.36	(0.23)	1.15***	(0.31)
Both partners employed in 2015			-0.14	(0.11)	-0.05	(0.11)	-0.01	(0.15)
Both employed in 2015, one not empl. in 2018			-0.05	(0.16)	-0.19	(0.16)	0.05	(0.22)
One not empl. in 2015, both empl. in 2018			-0.27*	(0.16)	-0.15	(0.16)	-0.71***	(0.22)
Received bequest			0.38**	(0.15)	-0.06	(0.15)	0.87***	(0.20)
Gave bequest			0.09	(0.40)	-0.04	(0.41)	-0.15	(0.56)
No child			*Ref.*		*Ref.*		*Ref.*	
1 child			-0.14	(0.11)	0.05	(0.12)	0.05	(0.16)
2 children			-0.12	(0.12)	-0.10	(0.12)	-0.31*	(0.17)
3 children or more			-0.00	(0.15)	0.02	(0.16)	-0.33	(0.21)
At least 1 child left home (2015-2018)			0.24*	(0.13)	0.32**	(0.13)	-0.20	(0.18)
Birth of a child (2015-2018)			-0.01	(0.14)	-0.21	(0.14)	-0.47**	(0.19)
Observations	1666		1666		1666		1666	
R^2	0.001		0.048		0.087		0.073	

Data: *Patrimoine* surveys 2014–2015 and 2017–2018.

Note: Standard errors in parentheses. * p<.1, ** p<.05, *** p<.01. Wealth is expressed in 1,000 euros (2015), IHS transformed, and constructed as follows. Net wealth: the sum of all the couple's assets minus liabilities (excluding business assets). Net housing wealth: the sum of all the couple's housing assets minus housing debt. Net financial wealth: the sum of all the couple's financial assets minus non-housing debt.

Table B.3: Legal status and gross wealth accumulation

	(1) Gross wealth		(2) Gross wealth		(3) Gross wealth	
Unregist. cohab.	0.173**	(0.079)	-0.053	(0.089)	-0.096	(0.088)
PACS	0.080	(0.101)	-0.176	(0.108)	-0.156	(0.106)
Married	*Ref.*		*Ref.*		*Ref.*	
Birth cohort (man): 1950–1959			0.008	(0.110)	0.000	(0.108)
Birth cohort (man): 1960–1969			*Ref.*		*Ref.*	
Birth cohort (man): 1970–1979			0.311***	(0.092)	0.345***	(0.090)
Birth cohort (man): 1980 or aft.			0.673***	(0.125)	0.686***	(0.123)
Age diff: M-F≥10			0.065	(0.156)	0.061	(0.153)
Age diff: M-F∈[3,10[			-0.036	(0.067)	-0.065	(0.066)
Age diff: M-F∈[-2,3[			*Ref.*		*Ref.*	
Age diff: M-F<-2			0.075	(0.099)	0.039	(0.097)
Couple form.: bef. 1980			*Ref.*		*Ref.*	
Couple form.: 1980–1989			0.220	(0.142)	0.190	(0.140)
Couple form.: 1990–1999			0.102	(0.162)	0.074	(0.159)
Couple form.: 2000–2009			0.343*	(0.176)	0.371**	(0.173)
Couple form.: 2010 or aft.			0.436**	(0.202)	0.398**	(0.199)
Rural			*Ref.*		*Ref.*	
Less than 20,000			0.126	(0.083)	0.126	(0.082)
20,000-100,000			-0.002	(0.100)	0.004	(0.098)
More than 100,000 (excl. Paris)			0.100	(0.080)	0.110	(0.079)
Paris and suburbs			0.307***	(0.099)	0.345***	(0.097)
Ever married			0.152*	(0.091)	0.186**	(0.089)
Edu (man): Low (ISCED 1–2)			*Ref.*		*Ref.*	
Edu (man): Medium (ISCED 3–4)			-0.228***	(0.083)	-0.213***	(0.082)
Edu (man): High (ISCED 5–8)			-0.087	(0.074)	-0.033	(0.073)
Edu (woman): Low (ISCED 1–2)			*Ref.*		*Ref.*	
Edu (woman): Medium (ISCED 3–4)			0.085	(0.083)	0.078	(0.082)
Edu (woman): High (ISCED 5–8)			-0.043	(0.079)	0.005	(0.078)
Chg in hh income (2015–2018)			0.003	(0.023)	0.019	(0.022)
Log of hh income (2015)			0.039	(0.028)	0.060**	(0.027)
Self-emp. in 2015			0.199***	(0.073)	0.235***	(0.072)
Self-emp. in 2015, not in 2018			-0.511**	(0.245)	-0.537**	(0.241)
Not self-emp. in 2015, self-emp. in 2018			-0.329*	(0.173)	-0.254	(0.171)
Both partners employed in 2015			-0.052	(0.081)	-0.004	(0.080)
Both employed in 2015, one not empl. in 2018			-0.164	(0.120)	-0.201*	(0.118)
One not empl. in 2015, both empl. in 2018			-0.164	(0.125)	-0.150	(0.123)
Received bequest			0.126	(0.113)	0.113	(0.111)
Gave bequest			0.057	(0.312)	0.002	(0.307)
0 child			*Ref.*		*Ref.*	
1 child			-0.158*	(0.088)	-0.119	(0.087)
2 children			-0.125	(0.092)	-0.081	(0.091)
3 children or more			0.011	(0.118)	0.041	(0.116)
At least 1 child left home (2015-2018)			0.233**	(0.101)	0.229**	(0.099)
Birth of a child (2015-2018)			-0.066	(0.107)	-0.050	(0.105)
Debt ×1,000 (2015)					-0.002***	(0.000)
Observations	1666		1666		1666	
R^2	0.003		0.084		0.115	

Data: *Patrimoine* surveys 2014–2015 and 2017–2018.
Note: Standard errors in parentheses. * p<.1, ** p<.05, *** p<.01. Wealth is expressed in 1,000 euros (2015) and IHS transformed. Gross wealth is constructed as the sum of all the couple's assets (excluding business assets).

Table B.4: Property regime and net wealth accumulation (full table)

	(1) Net wealth		(2) Net wealth		(3) Net housing wealth		(4) Net financial wealth	
Comm. property	*Ref.*		*Ref.*		*Ref.*		*Ref.*	
Sep. property	0.21***	(0.08)	0.03	(0.09)	0.06	(0.09)	0.19	(0.13)
Birth cohort (man): 1950–59			-0.12	(0.14)	-0.04	(0.14)	-0.19	(0.20)
Birth cohort (man): 1960–69			*Ref.*		*Ref.*		*Ref.*	
Birth cohort (man): 1970–79			0.27**	(0.12)	0.34***	(0.12)	0.00	(0.16)
Birth cohort (man): 1980 or aft.			0.37**	(0.16)	0.97***	(0.16)	-0.39*	(0.22)
Age diff: M-F$\geq$10			-0.24	(0.20)	0.19	(0.21)	-0.07	(0.28)
Age diff: M-F$\in$[3,10[			-0.08	(0.09)	0.07	(0.09)	-0.01	(0.12)
Age diff: M-F$\in$[-2,3[			*Ref.*		*Ref.*		*Ref.*	
Age diff: M-F<-2			-0.01	(0.13)	-0.07	(0.13)	0.12	(0.18)
Couple form.: bef 1980			*Ref.*		*Ref.*		*Ref.*	
Couple form.: 1980–89			0.21	(0.18)	0.21	(0.19)	0.02	(0.26)
Couple form.: 1990–99			0.17	(0.21)	0.15	(0.21)	-0.44	(0.29)
Couple form.: 2000–09			0.40*	(0.23)	0.37	(0.23)	-0.37	(0.32)
Couple form.: 2010 or aft.			0.53**	(0.26)	0.23	(0.26)	-0.17	(0.36)
Rural			*Ref.*		*Ref.*		*Ref.*	
Less than 20,0000			-0.05	(0.11)	0.03	(0.11)	0.19	(0.15)
20,000-100,000			-0.08	(0.13)	-0.19	(0.13)	0.29	(0.18)
More than 100,000 (excl. Paris)			0.09	(0.10)	0.04	(0.11)	0.19	(0.14)
Paris and suburbs			0.15	(0.13)	0.31**	(0.13)	-0.14	(0.18)
Ever married			0.10	(0.12)	0.10	(0.12)	-0.18	(0.16)
Edu (man): Low (ISCED 1–2)			*Ref.*		*Ref.*		*Ref.*	
Edu (man): Medium (ISCED 3–4)			-0.21*	(0.11)	-0.33***	(0.11)	0.31**	(0.15)
Edu (man): High (ISCED 5–8)			-0.15	(0.10)	-0.11	(0.10)	-0.12	(0.13)
Edu (woman): Low (ISCED 1–2)			*Ref.*		*Ref.*		*Ref.*	
Edu (woman): Medium (ISCED 3–4)			-0.02	(0.11)	0.04	(0.11)	-0.14	(0.15)
Edu (woman): High (ISCED 5–8)			-0.04	(0.10)	0.07	(0.10)	0.01	(0.14)
Chg in hh income (2015–2018)			0.01	(0.03)	0.06**	(0.03)	0.01	(0.04)
Log of hh income (2015)			0.08**	(0.04)	0.09**	(0.04)	0.08	(0.05)
Self-emp. in 2015			0.26***	(0.10)	0.41***	(0.10)	-0.11	(0.13)
Self-emp. in 2015, not in 2018			-0.54*	(0.32)	-0.68**	(0.32)	-0.21	(0.44)
Not self-emp. in 2015, self-emp. in 2018			-0.09	(0.22)	-0.37	(0.23)	1.16***	(0.31)
Both partners employed in 2015			-0.13	(0.10)	-0.06	(0.11)	0.00	(0.15)
Both employed in 2015, one not empl. in 2018			-0.06	(0.16)	-0.19	(0.16)	0.05	(0.22)
One not empl. in 2015, both empl. in 2018			-0.25	(0.16)	-0.15	(0.16)	-0.70***	(0.22)
Received bequest			0.37**	(0.15)	-0.06	(0.15)	0.85***	(0.20)
Gave bequest			0.09	(0.40)	-0.05	(0.41)	-0.09	(0.56)
No child			*Ref.*		*Ref.*		*Ref.*	
1 child			-0.14	(0.11)	0.05	(0.12)	0.05	(0.16)
2 children			-0.11	(0.12)	-0.10	(0.12)	-0.30*	(0.17)
3 children or more			0.01	(0.15)	0.02	(0.15)	-0.33	(0.21)
At least 1 child left home (2015-2018)			0.24*	(0.13)	0.32**	(0.13)	-0.20	(0.18)
Birth of a child (2015-2018)			-0.01	(0.14)	-0.22	(0.14)	-0.45**	(0.19)
Observations	1666		1666		1666		1666	
R^2	0.004		0.047		0.087		0.072	

Data: *Patrimoine* surveys 2014–2015 and 2017–2018.
Note: Standard errors in parentheses. * p<.1, ** p<.05, *** p<.01. Wealth is expressed in 1,000 euros (2015), IHS transformed, and constructed as follows. Net wealth: the sum of all the couple's assets minus liabilities (excluding business assets). Net housing wealth: the sum of all the couple's housing assets minus housing debt. Net financial wealth: the sum of all the couple's financial assets minus non-housing debt.

Table B.5: Property regime and gross wealth accumulation

	(1) Gross wealth		(2) Gross wealth		(3) Gross wealth	
Comm. property	*Ref.*		*Ref.*		*Ref.*	
Sep. property	0.15**	(0.06)	-0.05	(0.07)	-0.04	(0.07)
Birth cohort (man): 1950–59			0.01	(0.11)	0.01	(0.11)
Birth cohort (man): 1960–69			*Ref.*		*Ref.*	
Birth cohort (man): 1970–79			0.31***	(0.09)	0.34***	(0.09)
Birth cohort (man): 1980 or aft.			0.66***	(0.13)	0.68***	(0.12)
Age diff: M-F≥10			0.06	(0.16)	0.06	(0.15)
Age diff: M-F∈[3,10[			-0.04	(0.07)	-0.07	(0.07)
Age diff: M-F∈[-2,3[			*Ref.*		*Ref.*	
Age diff: M-F<-2			0.06	(0.10)	0.03	(0.10)
Couple form.: bef. 1980			*Ref.*		*Ref.*	
Couple form.: 1980-89			0.23	(0.14)	0.19	(0.14)
Couple form.: 1990-99			0.11	(0.16)	0.07	(0.16)
Couple form.: 2000-09			0.34*	(0.18)	0.35**	(0.17)
Couple form.: 2010 or aft.			0.43**	(0.20)	0.36*	(0.20)
Rural			*Ref.*		*Ref.*	
Less than 20,0000			0.12	(0.08)	0.12	(0.08)
20,000-100,000			-0.00	(0.10)	-0.00	(0.10)
More than 100,000 (excl. Paris)			0.09	(0.08)	0.10	(0.08)
Paris and suburbs			0.31***	(0.10)	0.34***	(0.10)
Ever married			0.15*	(0.09)	0.18**	(0.09)
Edu. (man): Low (ISCED 1–2)			*Ref.*		*Ref.*	
Edu. (man): Medium (ISCED 3–4)			-0.23***	(0.08)	-0.21***	(0.08)
Edu. (man): High (ISCED 5–8)			-0.09	(0.07)	-0.04	(0.07)
Edu. (woman): Low (ISCED 1–2)			*Ref.*		*Ref.*	
Edu. (woman): Medium (ISCED 3–4)			0.09	(0.08)	0.08	(0.08)
Edu. (woman): High (ISCED 5–8)			-0.04	(0.08)	0.01	(0.08)
Chg in hh income (2018-2015)			0.00	(0.02)	0.02	(0.02)
Log of hh income (2015)			0.04	(0.03)	0.06**	(0.03)
Self-emp. in 2015			0.21***	(0.07)	0.24***	(0.07)
Self-emp. in 2015, not in 2018			-0.50**	(0.25)	-0.53**	(0.24)
Not self-emp. in 2015, self-emp. in 2018			-0.33*	(0.17)	-0.25	(0.17)
Both partners employed in 2015			-0.06	(0.08)	-0.01	(0.08)
Both employed in 2015, one not empl. in 2018			-0.16	(0.12)	-0.20*	(0.12)
One not empl. in 2015, both empl. in 2018			-0.16	(0.12)	-0.14	(0.12)
Received bequest			0.13	(0.11)	0.11	(0.11)
Gave bequest			0.03	(0.31)	-0.02	(0.31)
0 child			*Ref.*		*Ref.*	
1 child			-0.16*	(0.09)	-0.12	(0.09)
2 children			-0.13	(0.09)	-0.08	(0.09)
3 children or more			0.02	(0.12)	0.05	(0.12)
At least 1 child left home (2015-2018)			0.24**	(0.10)	0.23**	(0.10)
Birth of a child (2015-2018)			-0.07	(0.11)	-0.05	(0.11)
Debt ×1,000 (2015)					-0.00***	(0.00)
Observations	1666		1666		1666	
R^2	0.004		0.083		0.113	

Data: *Patrimoine* surveys 2014–2015 and 2017–2018.
Note: Standard errors in parentheses. * p<.1, ** p<.05, *** p<.01. Wealth is expressed in 1,000 euros (2015) and IHS transformed. Gross wealth is constructed as the sum of all the couple's assets (excluding business assets).

Table B.6: Marital status and net wealth accumulation (full table)

	(1) Net wealth		(2) Net wealth		(3) Net housing wealth		(4) Net financial wealth	
Unregist. cohab.	0.14	(0.10)	-0.09	(0.12)	0.09	(0.12)	0.11	(0.16)
Married (comm. assets)	*Ref.*		*Ref.*		*Ref.*		*Ref.*	
Married (sep. assets)	0.40***	(0.14)	0.32**	(0.15)	0.16	(0.15)	0.15	(0.20)
PACS (comm. assets)	0.17	(0.27)	0.13	(0.28)	0.35	(0.28)	0.26	(0.39)
PACS (sep. assets)	0.18	(0.14)	-0.08	(0.16)	-0.10	(0.16)	0.45**	(0.22)
Birth cohort (man): 1950–1959			-0.15	(0.14)	-0.05	(0.14)	-0.19	(0.20)
Birth cohort (man): 1960–1969			*Ref.*		*Ref.*		*Ref.*	
Birth cohort (man): 1970–1979			0.27**	(0.12)	0.34***	(0.12)	-0.01	(0.16)
Birth cohort (man): 1980 or aft.			0.40**	(0.16)	0.99***	(0.17)	-0.41*	(0.23)
Age diff: M-F≥10			-0.24	(0.20)	0.20	(0.21)	-0.08	(0.28)
Age diff: M-F∈[3,10[			-0.07	(0.09)	0.08	(0.09)	-0.02	(0.12)
Age diff: M-F∈[-2,3[			*Ref.*		*Ref.*		*Ref.*	
Age diff: M-F<-2			0.01	(0.13)	-0.06	(0.13)	0.10	(0.18)
Couple form.: bef. 1980			*Ref.*		*Ref.*		*Ref.*	
Couple form.: 1980–1989			0.21	(0.18)	0.20	(0.19)	0.03	(0.26)
Couple form.: 1990–1999			0.18	(0.21)	0.14	(0.21)	-0.41	(0.29)
Couple form.: 2000–2009			0.42*	(0.23)	0.36	(0.23)	-0.36	(0.32)
Couple form.: 2010 or aft.			0.59**	(0.26)	0.22	(0.27)	-0.15	(0.36)
Rural			*Ref.*		*Ref.*		*Ref.*	
Less than 20,0000			-0.04	(0.11)	0.03	(0.11)	0.18	(0.15)
20,000-100,000			-0.09	(0.13)	-0.20	(0.13)	0.28	(0.18)
More than 100,000 (excl. Paris)			0.10	(0.10)	0.05	(0.11)	0.17	(0.14)
Paris and suburbs			0.16	(0.13)	0.31**	(0.13)	-0.14	(0.18)
Ever married			0.09	(0.12)	0.09	(0.12)	-0.18	(0.16)
Edu. (man): Low (ISCED 1–2)			*Ref.*		*Ref.*		*Ref.*	
Edu. (man): Medium (ISCED 3–4)			-0.22**	(0.11)	-0.33***	(0.11)	0.30**	(0.15)
Edu. (man): High (ISCED 5–8)			-0.16*	(0.10)	-0.11	(0.10)	-0.15	(0.13)
Edu. (woman): Low (ISCED 1–2)			*Ref.*		*Ref.*		*Ref.*	
Edu. (woman): Medium (ISCED 3–4)			-0.04	(0.11)	0.04	(0.11)	-0.14	(0.15)
Edu. (woman): High (ISCED 5–8)			-0.07	(0.10)	0.06	(0.10)	0.01	(0.14)
Chg. in hh income (2018-2015)			0.00	(0.03)	0.06**	(0.03)	0.00	(0.04)
Log of hh income (2015)			0.07**	(0.04)	0.09**	(0.04)	0.07	(0.05)
Self-emp. in 2015			0.22**	(0.10)	0.39***	(0.10)	-0.10	(0.13)
Self-emp. in 2015, not in 2018			-0.54*	(0.32)	-0.68**	(0.32)	-0.19	(0.44)
Not self-emp. in 2015, self-emp. in 2018			-0.09	(0.22)	-0.34	(0.23)	1.14***	(0.31)
Both partners employed in 2015			-0.14	(0.11)	-0.06	(0.11)	-0.01	(0.15)
Both employed in 2015, one not empl. in 2018			-0.04	(0.16)	-0.18	(0.16)	0.05	(0.22)
One not empl. in 2015, both empl. in 2018			-0.26	(0.16)	-0.15	(0.16)	-0.70***	(0.22)
Received bequest			0.38***	(0.15)	-0.06	(0.15)	0.86***	(0.20)
Gave bequest			0.05	(0.41)	-0.11	(0.41)	-0.13	(0.56)
No child			*Ref.*		*Ref.*		*Ref.*	
1 child			-0.15	(0.11)	0.04	(0.12)	0.05	(0.16)
2 children			-0.14	(0.12)	-0.12	(0.12)	-0.31*	(0.17)
3 children or more			-0.01	(0.15)	0.01	(0.16)	-0.33	(0.21)
At least 1 child left home (2015–2018)			0.25*	(0.13)	0.33**	(0.13)	-0.20	(0.18)
Birth of a child (2015–2018)			-0.03	(0.14)	-0.24*	(0.14)	-0.46**	(0.19)
Observations	1666		1666		1666		1666	
R^2	0.006		0.051		0.089		0.074	

Data: *Patrimoine* surveys 2014–2015 and 2017–2018.
Note: Standard errors in parentheses. * p<.1, ** p<.05, *** p<.01. Wealth is expressed in 1,000 euros (2015), IHS transformed, and constructed as follows. Net wealth: the sum of all the couple's assets minus liabilities (excluding business assets). Net housing wealth: the sum of all the couple's housing assets minus housing debt. Net financial wealth: the sum of all the couple's financial assets minus non-housing debt.

Table B.7: Marital status and gross wealth accumulation

	(1) Gross wealth		(2) Gross wealth		(3) Gross wealth	
Unregist. cohab.	0.19**	(0.08)	-0.05	(0.09)	-0.08	(0.09)
Married (comm. assets)	*Ref.*		*Ref.*		*Ref.*	
Married (sep. assets)	0.14	(0.11)	0.05	(0.11)	0.13	(0.11)
Pacs (comm. assets)	0.06	(0.21)	-0.03	(0.21)	0.01	(0.21)
Pacs (sep. assets)	0.10	(0.11)	-0.21*	(0.12)	-0.18	(0.12)
Birth cohort (man): 1950–1959			0.00	(0.11)	-0.01	(0.11)
Birth cohort (man): 1960–1969			*Ref.*		*Ref.*	
Birth cohort (man): 1970–1979			0.31***	(0.09)	0.35***	(0.09)
Birth cohort (man): 1980 or aft.			0.68***	(0.13)	0.70***	(0.12)
Age diff: M-F$\geq$10			0.07	(0.16)	0.06	(0.15)
Age diff: M-F$\in$[3,10[			-0.03	(0.07)	-0.06	(0.07)
Age diff: M-F$\in$[-2,3[			*Ref.*		*Ref.*	
Age diff: M-F$<$-2			0.08	(0.10)	0.04	(0.10)
Couple form.: bef. 1980			*Ref.*		*Ref.*	
Couple form.: 1980–1989			0.22	(0.14)	0.18	(0.14)
Couple form.: 1990–1999			0.10	(0.16)	0.06	(0.16)
Couple form.: 2000–2009			0.34*	(0.18)	0.36**	(0.17)
Couple form.: 2010 or aft.			0.44**	(0.20)	0.39*	(0.20)
Rural			*Ref.*		*Ref.*	
Less than 20,0000			0.13	(0.08)	0.13	(0.08)
20,000-100,000			-0.01	(0.10)	-0.00	(0.10)
More than 100,000 (excl. Paris)			0.10	(0.08)	0.11	(0.08)
Paris and suburbs			0.31***	(0.10)	0.35***	(0.10)
Ever married			0.15	(0.09)	0.18**	(0.09)
Edu. (man): Low (ISCED 1–2)			*Ref.*		*Ref.*	
Edu. (man): Medium (ISCED 3–4)			-0.23***	(0.08)	-0.21***	(0.08)
Edu. (man): High (ISCED 5–8)			-0.09	(0.07)	-0.04	(0.07)
Edu. (woman): Low (ISCED 1–2)			*Ref.*		*Ref.*	
Edu. (woman): Medium (ISCED 3–4)			0.08	(0.08)	0.07	(0.08)
Edu. (woman): High (ISCED 5–8)			-0.05	(0.08)	-0.00	(0.08)
Chg. in hh income (2018-2015)			0.00	(0.02)	0.02	(0.02)
Log of hh income (2015)			0.04	(0.03)	0.06**	(0.03)
Self-emp. in 2015			0.19***	(0.07)	0.22***	(0.07)
Self-emp. in 2015, not in 2018			-0.51**	(0.25)	-0.54**	(0.24)
Not self-emp. in 2015, self-emp. in 2018			-0.32*	(0.17)	-0.25	(0.17)
Both partners employed in 2015			-0.05	(0.08)	-0.01	(0.08)
Both employed in 2015, one not empl. in 2018			-0.16	(0.12)	-0.19*	(0.12)
One not empl. in 2015, both empl. in 2018			-0.16	(0.12)	-0.14	(0.12)
Received bequest			0.13	(0.11)	0.12	(0.11)
Gave bequest			0.03	(0.31)	-0.03	(0.31)
No child			*Ref.*		*Ref.*	
1 child			-0.16*	(0.09)	-0.13	(0.09)
2 children			-0.13	(0.09)	-0.09	(0.09)
3 children or more			0.01	(0.12)	0.04	(0.12)
At least 1 child left home (2015–2018)			0.24**	(0.10)	0.24**	(0.10)
Birth of a child (2015–2018)			-0.07	(0.11)	-0.06	(0.11)
Debt $\times$1,000 (2015)					-0.00***	(0.00)
Observations	1666		1666		1666	
R^2	0.004		0.085		0.116	

Data: *Patrimoine* surveys 2014–2015 and 2017–2018.
Note: Standard errors in parentheses. * p<.1, ** p<.05, *** p<.01. Wealth is expressed in 1,000 euros (2015) and IHS transformed. Gross wealth is constructed as the sum of all the couple's assets (excluding business assets).

Table B.8: Marital status and female partner's share of household net wealth (full table)

	(1) Female share		(2) Female share		(3) Female share		(4) Female share	
Cohab.	0.011	(0.012)	0.015	(0.014)	0.090***	(0.015)	-0.038**	(0.016)
Married (comm.)	*Ref.*		*Ref.*		*Ref.*		*Ref.*	
Married (sep.)	0.023	(0.016)	0.023	(0.017)	0.062***	(0.018)	-0.042*	(0.022)
PACS (comm.)	-0.000	(0.030)	-0.007	(0.031)	-0.016	(0.031)	0.005	(0.038)
PACS (sep.)	0.001	(0.016)	-0.003	(0.018)	0.059***	(0.019)	-0.066***	(0.021)
Birth cohort (man): 1950–1959			0.004	(0.016)	0.008	(0.015)	0.005	(0.016)
Birth cohort (man): 1960–1969			*Ref.*		*Ref.*		*Ref.*	
Birth cohort (man): 1970–1979			0.021	(0.014)	0.022*	(0.013)	0.028**	(0.013)
Birth cohort (man): 1980 or aft.			0.035*	(0.019)	0.032*	(0.018)	0.046**	(0.018)
Age diff: M-F≥10			0.040*	(0.024)	0.049**	(0.022)	0.060***	(0.022)
Age diff: M-F∈[3,10[			0.016	(0.010)	0.006	(0.009)	0.016*	(0.009)
Age diff: M-F∈[-2,3[			*Ref.*		*Ref.*		*Ref.*	
Age diff: M-F<-2			0.026*	(0.015)	0.042***	(0.014)	0.027*	(0.015)
Couple form.: bef. 1980			*Ref.*		*Ref.*		*Ref.*	
Couple form.: 1980–1989			-0.021	(0.021)	-0.014	(0.020)	-0.021	(0.020)
Couple form.: 1990–1999			-0.037	(0.024)	-0.035	(0.023)	-0.054**	(0.023)
Couple form.: 2000–2009			-0.060**	(0.027)	-0.052**	(0.025)	-0.073***	(0.025)
Couple form.: 2010 or aft.			-0.033	(0.031)	-0.033	(0.029)	-0.064**	(0.029)
Rural			*Ref.*		*Ref.*		*Ref.*	
Less than 20,0000			0.003	(0.012)	0.008	(0.012)	0.008	(0.012)
20,000-100,000			0.001	(0.015)	-0.003	(0.014)	0.002	(0.014)
More than 100,000 (excl. Paris)			0.019	(0.012)	0.011	(0.011)	0.011	(0.012)
Paris and suburbs			0.010	(0.015)	0.002	(0.014)	0.004	(0.015)
Ever married			0.017	(0.014)	0.031**	(0.013)	0.021	(0.013)
Edu. (man): Low (ISCED 1–2)			*Ref.*		*Ref.*		*Ref.*	
Edu. (man): Medium (ISCED 3–4)			0.008	(0.012)	0.000	(0.012)	-0.004	(0.012)
Edu. (man): High (ISCED 5–8)			-0.018	(0.011)	-0.017*	(0.010)	-0.023**	(0.011)
Edu. (woman): Low (ISCED 1–2)			*Ref.*		*Ref.*		*Ref.*	
Edu. (woman): Medium (ISCED 3–4)			0.026**	(0.013)	0.027**	(0.012)	0.023*	(0.012)
Edu. (woman): High (ISCED 5–8)			0.019	(0.012)	0.031***	(0.011)	0.019*	(0.011)
Chg. in hh income (2018-2015)			-0.007**	(0.004)	-0.009***	(0.003)	-0.008**	(0.003)
Log of hh income (2015)			-0.003	(0.005)	-0.003	(0.004)	0.003	(0.004)
Self-emp. in 2015			-0.003	(0.011)	-0.009	(0.010)	-0.016	(0.011)
Self-emp. in 2015, not in 2018			0.087**	(0.039)	0.108***	(0.037)	0.079**	(0.037)
Not self-emp. in 2015, self-emp. in 2018			-0.001	(0.025)	-0.029	(0.024)	-0.014	(0.024)
Both partners employed in 2015			0.031**	(0.012)	0.029**	(0.012)	0.016	(0.012)
Both employed in 2015, one not empl. in 2018			-0.015	(0.018)	-0.023	(0.017)	-0.019	(0.017)
One not empl. in 2015, both empl. in 2018			0.033*	(0.019)	0.043**	(0.018)	0.022	(0.018)
Received bequest			-0.023	(0.017)	-0.010	(0.016)	-0.009	(0.016)
Gave bequest			0.054	(0.046)	0.053	(0.044)	0.046	(0.044)
No child			*Ref.*		*Ref.*		*Ref.*	
1 child			-0.017	(0.013)	-0.018	(0.012)	-0.016	(0.013)
2 children			-0.001	(0.014)	0.003	(0.013)	0.019	(0.013)
3 children or more			0.013	(0.018)	0.014	(0.017)	0.035**	(0.017)
At least 1 kid left home (2015-2018)			0.009	(0.015)	0.003	(0.014)	0.009	(0.014)
Birth of a child (2015-2018)			0.012	(0.016)	0.010	(0.015)	0.015	(0.015)
F richer than M					-0.125***	(0.022)		
Cohab. × F richer than M					-0.096***	(0.030)		
Married (sep.) × F richer than M					-0.093**	(0.041)		
PACS (comm.) × F richer than M					0.101	(0.090)		
PACS (sep.) × F richer than M					-0.126***	(0.039)		
F inc. sh. > F W. sh.							0.054***	(0.011)
Cohab. × F inc. sh. > F W. sh.							0.136***	(0.024)
Married (sep.) × F inc. sh. > F W. sh.							0.112***	(0.031)
PACS (comm.) × F inc. sh. > F W. sh.							-0.038	(0.060)
PACS (sep.) × F inc. sh. > F W. sh.							0.159***	(0.033)
Constant	0.001	(0.005)	0.013	(0.053)	0.008	(0.049)	-0.057	(0.051)
Observations	1612		1612		1612		1612	
R^2	0.002		0.037		0.166		0.135	

Data: *Patrimoine* surveys 2014–2015 and 2017–2018.

Note: Standard errors in parentheses. * p<.1, ** p<.05, *** p<.01. Female partner's share is her share of the net household wealth. Net wealth is constructed as the sum of all the couple's assets minus liabilities (excluding business assets).

Table B.9: Marital status and changes in female partner's share of household gross wealth (2015–2018)

	(1) Female share		(2) Female share		(3) Female share		(4) Female share	
Cohab.	0.018	(0.011)	0.020	(0.013)	0.088***	(0.014)	-0.022	(0.015)
Married (comm.)	*Ref.*		*Ref.*		*Ref.*		*Ref.*	
Married (sep.)	0.030**	(0.015)	0.026*	(0.016)	0.049***	(0.017)	-0.014	(0.021)
PACS (comm.)	0.003	(0.029)	-0.005	(0.029)	-0.014	(0.030)	0.008	(0.036)
PACS (sep.)	0.018	(0.015)	0.014	(0.017)	0.056***	(0.018)	-0.041**	(0.020)
Birth cohort (man): 1950–59			-0.002	(0.015)	0.002	(0.015)	-0.002	(0.015)
Birth cohort (man): 1960–69			*Ref.*		*Ref.*		*Ref.*	
Birth cohort (man): 1970–79			0.016	(0.013)	0.018	(0.012)	0.022*	(0.013)
Birth cohort (man): 1980 or aft.			0.029	(0.018)	0.025	(0.017)	0.039**	(0.017)
Age diff: M-F$\geq$10			0.050**	(0.022)	0.052**	(0.021)	0.066***	(0.021)
Age diff: M-F$\in$[3,10[			0.018*	(0.009)	0.008	(0.009)	0.018**	(0.009)
Age diff: M-F$\in$[-2,3[			*Ref.*		*Ref.*		*Ref.*	
Age diff: M-F<-2			0.030**	(0.014)	0.044***	(0.014)	0.030**	(0.014)
Couple form.: bef. 1980			*Ref.*		*Ref.*		*Ref.*	
Couple form.: 1980–1989			-0.025	(0.020)	-0.016	(0.019)	-0.025	(0.019)
Couple form.: 1990–1999			-0.044*	(0.023)	-0.040*	(0.022)	-0.058***	(0.022)
Couple form.: 2000–2009			-0.061**	(0.025)	-0.049**	(0.024)	-0.071***	(0.024)
Couple form.: 2010 or aft.			-0.030	(0.029)	-0.025	(0.027)	-0.056**	(0.028)
Rural			*Ref.*		*Ref.*		*Ref.*	
Less than 20,0000			0.008	(0.012)	0.013	(0.011)	0.013	(0.011)
20,000-100,000			0.006	(0.014)	0.003	(0.013)	0.007	(0.013)
More than 100,000 (excl. Paris)			0.020*	(0.011)	0.014	(0.011)	0.014	(0.011)
Paris and suburbs			0.008	(0.014)	0.004	(0.014)	0.003	(0.014)
Ever married			0.019	(0.013)	0.031**	(0.013)	0.022*	(0.013)
Edu. (man): Low (ISCED 1–2)			*Ref.*		*Ref.*		*Ref.*	
Edu. (man): Medium (ISCED 3-4)			0.014	(0.012)	0.007	(0.011)	0.004	(0.011)
Edu. (man): High (ISCED 5-8)			-0.011	(0.010)	-0.010	(0.010)	-0.016	(0.010)
Edu. (woman): Low (ISCED 1–2)			*Ref.*		*Ref.*		*Ref.*	
Edu. (woman): Medium (ISCED 3-4)			0.022*	(0.012)	0.022**	(0.011)	0.020*	(0.011)
Edu. (woman): High (ISCED 5-8)			0.019*	(0.011)	0.030***	(0.010)	0.020*	(0.011)
Chg. in hh income (2018-2015)			-0.009***	(0.003)	-0.010***	(0.003)	-0.010***	(0.003)
Log of hh income (2015)			-0.004	(0.004)	-0.002	(0.004)	0.002	(0.004)
Self-emp. in 2015			0.004	(0.010)	0.001	(0.010)	-0.006	(0.010)
Self-emp. in 2015, not in 2018			0.078**	(0.037)	0.098***	(0.035)	0.071**	(0.035)
Not self-emp. in 2015, self-emp. in 2018			-0.013	(0.024)	-0.034	(0.023)	-0.024	(0.023)
Both partners employed in 2015			0.024**	(0.012)	0.020*	(0.011)	0.010	(0.011)
Both employed in 2015, one not empl. in 2018			-0.010	(0.017)	-0.017	(0.016)	-0.013	(0.016)
One not empl. in 2015, both empl. in 2018			0.024	(0.018)	0.032*	(0.017)	0.014	(0.017)
Received bequest			-0.027*	(0.016)	-0.016	(0.015)	-0.016	(0.015)
Gave bequest			0.051	(0.043)	0.051	(0.042)	0.046	(0.041)
No child			*Ref.*		*Ref.*		*Ref.*	
1 child			-0.009	(0.012)	-0.008	(0.012)	-0.007	(0.012)
2 children			0.006	(0.013)	0.011	(0.012)	0.023*	(0.013)
3 children or more			0.017	(0.017)	0.020	(0.016)	0.037**	(0.016)
At least 1 kid left home (2015–2018)			0.002	(0.014)	-0.004	(0.013)	0.003	(0.014)
Birth of a child (2015–2018)			0.004	(0.015)	0.003	(0.014)	0.006	(0.015)
F richer than M					-0.125***	(0.021)		
Cohab. × F richer than M					-0.080***	(0.029)		
Married (sep.) × F richer than M					-0.009	(0.038)		
PACS (comm.) × F richer than M					0.110	(0.085)		
PACS (sep.) × F richer than M					-0.053	(0.037)		
F inc. sh. > F W. sh.							0.052***	(0.011)
Cohab. × F inc. sh. > F W. sh.							0.106***	(0.023)
Married (sep.) × F inc. sh. > F W. sh.							0.067**	(0.029)
PACS (comm.) × F inc. sh. > F W. sh.							-0.039	(0.057)
PACS (sep.) × F inc. sh. > F W. sh.							0.137***	(0.031)
Debt ×1,000 (2015)			-0.000	(0.000)	-0.000	(0.000)	-0.000	(0.000)
Constant	-0.001	(0.005)	0.018	(0.050)	0.004	(0.047)	-0.047	(0.048)
Observations	1615		1615		1615		1615	
R^2	0.004		0.046		0.149		0.126	

Data: *Patrimoine* surveys 2014–2015 and 2017–2018.
Note: Standard errors in parentheses. * p<.1, ** p<.05, *** p<.01. Female partner's share is her share of the gross household wealth. Gross wealth is constructed as the sum of all the couple's assets (excluding business assets).

Table B.10: Marital status and changes the female partner's and male partner's net wealth (2015–2018)

	(1) Female net wealth		(2) Female net wealth		(3) Male net wealth		(4) Male net wealth	
Cohab.	0.136	(0.100)	-0.002	(0.115)	0.216**	(0.095)	-0.021	(0.108)
Married (comm.)	*Ref.*		*Ref.*		*Ref.*		*Ref.*	
Married (sep.)	0.416***	(0.138)	0.310**	(0.145)	0.285**	(0.131)	0.249*	(0.137)
PACS (comm.)	0.159	(0.267)	0.131	(0.274)	0.163	(0.253)	0.130	(0.257)
PACS (sep.)	0.070	(0.140)	-0.150	(0.153)	0.224*	(0.133)	-0.015	(0.144)
Birth cohort (man): 1950–1959			-0.155	(0.140)			-0.025	(0.132)
Birth cohort (man): 1960–1969			*Ref.*				*Ref.*	
Birth cohort (man): 1970–1979			0.320***	(0.117)			0.299***	(0.110)
Birth cohort (man): 1980 or aft.			0.730***	(0.160)			0.273*	(0.151)
Age diff: M-F≥10			0.070	(0.199)			-0.568***	(0.187)
Age diff: M-F∈[3,10[			0.084	(0.086)			-0.054	(0.080)
Age diff: M-F∈[-2,3[			*Ref.*				*Ref.*	
Age diff: M-F<-2			0.004	(0.127)			0.005	(0.119)
Couple form.: bef. 1980			*Ref.*				*Ref.*	
Couple form.: 1980–1989			0.096	(0.181)			0.382**	(0.171)
Couple form.: 1990–1999			-0.020	(0.207)			0.364*	(0.195)
Couple form.: 2000–2009			0.017	(0.226)			0.549***	(0.212)
Couple form.: 2010 or aft.			0.083	(0.259)			0.816***	(0.243)
Rural			*Ref.*				*Ref.*	
Less than 20,0000			0.112	(0.107)			-0.079	(0.100)
20,000-100,000			0.066	(0.128)			-0.096	(0.120)
More than 100,000 (excl. Paris)			0.207**	(0.103)			0.060	(0.096)
Paris and suburbs			0.192	(0.126)			0.188	(0.119)
Ever married			0.120	(0.116)			0.025	(0.109)
Edu. (man): Low (ISCED 1–2)			*Ref.*				*Ref.*	
Edu. (man): Medium (ISCED 3–4)			0.020	(0.106)			-0.161	(0.100)
Edu. (man): High (ISCED 5–8)			-0.161*	(0.095)			-0.125	(0.089)
Edu. (woman): Low (ISCED 1–2)			*Ref.*				*Ref.*	
Edu. (woman): Medium (ISCED 3–4)			0.080	(0.106)			-0.065	(0.100)
Edu. (woman): High (ISCED 5–8)			-0.013	(0.101)			-0.107	(0.095)
Chg. in hh income (2018-2015)			-0.021	(0.029)			0.012	(0.027)
Log of hh income (2015)			0.043	(0.035)			0.080**	(0.033)
Self-emp. in 2015			0.349***	(0.095)			0.210**	(0.089)
Self-emp. in 2015, not in 2018			-0.249	(0.313)			-1.113***	(0.294)
Not self-emp. in 2015, self-emp. in 2018			0.188	(0.222)			-0.133	(0.208)
Both partners employed in 2015			0.038	(0.104)			-0.171*	(0.098)
Both employed in 2015, one not empl. in 2018			-0.101	(0.153)			0.090	(0.144)
One not empl. in 2015, both empl. in 2018			-0.173	(0.159)			-0.170	(0.150)
Received bequest			0.206	(0.144)			0.485***	(0.136)
Gave bequest			0.449	(0.401)			0.026	(0.377)
No child			*Ref.*				*Ref.*	
1 child			-0.166	(0.112)			-0.070	(0.106)
2 children			-0.178	(0.118)			-0.185*	(0.111)
3 children or more			-0.016	(0.151)			0.022	(0.142)
At least 1 child left home (2015–2018)			0.205	(0.129)			0.216*	(0.121)
Birth of a child (2015–2018)			-0.191	(0.137)			0.068	(0.129)
Constant	0.231***	(0.045)	-0.541	(0.414)	0.219***	(0.043)	-0.885**	(0.389)
Observations	1666		1666		1666		1666	
R^2	0.006		0.053		0.006		0.072	

Data: *Patrimoine* surveys 2014–2015 and 2017–2018.
Note: Standard errors in parentheses. * p<.1, ** p<.05, *** p<.01. Female partner's net wealth is the sum of all her assets and half of joint assets. Male partner's net wealth is the sum of all his assets and half of joint assets. Net wealth is constructed as the sum of all the couple's assets minus liabilities (excluding business assets).

Table B.11: Marital status and net wealth accumulation, couples formed between 2005 and 2015

	(1) Net wealth	(2) Net wealth	(3) Net wealth	(4) Net wealth	(5) Net housing wealth	(6) Net financial wealth
Cohab.	-0.067	-0.015	-0.018	-0.264	0.134	0.150
	(0.332)	(0.368)	(0.412)	(0.405)	(0.394)	(0.447)
Married (comm.)	*Ref.*	*Ref.*	*Ref.*	*Ref.*	*Ref.*	*Ref.*
Married (sep.)	1.189**	1.056*	1.230**	1.032*	0.606	0.549
	(0.542)	(0.562)	(0.599)	(0.588)	(0.572)	(0.648)
PACS (sep.)	0.139	0.353	0.496	0.612	0.107	0.654
	(0.361)	(0.396)	(0.424)	(0.414)	(0.404)	(0.457)
Demographics		X	X	X	X	X
Professional			X	X	X	X
Bequest				X	X	X
Observations	225	225	225	225	225	225
R^2	0.025	0.084	0.139	0.223	0.197	0.259

Data: *Patrimoine* surveys 2014–2015 and 2017–2018.
Note: Standard errors in parentheses. * p<.1, ** p<.05, *** p<.01. Demographic characteristics include: male partner's birth cohort, age difference between partners, year of couple formation, size of residence city, citizenship of both partners, and past marriage. Professional characteristics include: educational attainment of both partners, 2015 household income (in log), changes in household income (2015–2018), at least one partner is self-employed in 2015, change in self-employment (2015–2018), at least one partner is not working full-time in 2015, and change in full-time employment (2015–2018). Bequest characteristics include: dummy variable for receiving a bequest or a gift (2015–2018), dummy variable for giving an inter vivos gift (2015–2018), number of children in the household in 2015, binary for a decrease in the number of children (2015–2018), and binary for an increase in number of children (2015–2018).
Wealth is expressed in 1,000 euros (2015), IHS transformed, and constructed as follows. Net wealth: the sum of all the couple's assets minus liabilities (excluding business assets). Net housing wealth: the sum of all the couple's housing assets minus housing debt. Net financial wealth: the sum of all the couple's financial assets minus non-housing debt. Few recent couples with PACS couples with a community property regime were observed, the category was excluded.

Table B.12: Marital status and changes in female partner's share of household net wealth, couples formed between 2005 and 2015

	(1) Female share	(2) Female share	(3) Female share	(4) Female share	(5) Female share	(6) Female share
Cohab.	-0.005	0.000	0.005	0.005	0.171***	-0.111*
	(0.045)	(0.049)	(0.056)	(0.058)	(0.057)	(0.061)
Married (comm.)	*Ref.*	*Ref.*	*Ref.*	*Ref.*	*Ref.*	*Ref.*
Married (sep.)	0.188***	0.201***	0.212***	0.204**	0.251***	-0.066
	(0.071)	(0.073)	(0.078)	(0.079)	(0.073)	(0.135)
PACS (sep.)	0.044	0.059	0.079	0.099*	0.210***	-0.010
	(0.048)	(0.052)	(0.056)	(0.057)	(0.054)	(0.065)
F richer than M					-0.127	
					(0.135)	
Cohab. × F richer than M					-0.286*	
					(0.151)	
Married (sep.) × F richer than M					-0.223	
					(0.225)	
PACS (sep.) × F richer than M					-0.271*	
					(0.158)	
F inc. sh. > F W. sh.						0.104
						(0.066)
Cohab. × F inc. sh. > F W. sh.						0.257***
						(0.098)
Married (sep.) × F inc. sh. > F W. sh.						0.308*
						(0.164)
PACS (sep.) × F inc. sh. > F W. sh.						0.244**
						(0.103)
Constant	-0.005	-0.115	0.146	0.329	0.290	0.051
	(0.029)	(0.124)	(0.239)	(0.262)	(0.228)	(0.250)
Demographics		X	X	X	X	X
Professional			X	X	X	X
Bequest				X	X	X
Wealth gap between spouses					X	
Female wealth/income comparison						X
Observations	215	215	215	215	215	215
R^2	0.037	0.121	0.176	0.206	0.418	0.387

Data: *Patrimoine* surveys 2014–2015 and 2017–2018.
Note: Standard errors in parentheses. * p<.1, ** p<.05, *** p<.01. Demographic characteristics include: male partner's birth cohort, age difference between partners, year of couple formation, size of residence city, citizenship of both partners, and past marriage. Professional characteristics include: educational attainment of both partners, 2015 household income (in log), changes in household income (2015–2018), at least one partner is self-employed in 2015, change in self-employment (2015–2018), at least one partner is not working full-time in 2015, and change in full-time employment (2015–2018). Bequest characteristics include: dummy variable for receiving a bequest or a gift (2015–2018), dummy variable for giving an inter vivos gift (2015–2018), number of children in the household in 2015, binary for a decrease in the number of children (2015–2018), and binary for an increase in number of children (2015–2018). Female partner's share is her share of the net household wealth. Net wealth is constructed as the sum of all the couple's assets minus liabilities (excluding business assets).

Table B.13: Marital status and wealth accumulation, sample including couples transitioning across marital status (2015–2018)

	(1) Net wealth	(2) Net wealth	(3) Net wealth	(4) Net wealth	(5) Net housing wealth	(6) Net financial wealth
Cohab.	0.221**	-0.007	-0.053	-0.061	0.112	0.078
	(0.092)	(0.107)	(0.109)	(0.109)	(0.110)	(0.149)
Married (comm.)	*Ref.*	*Ref.*	*Ref.*	*Ref.*	*Ref.*	*Ref.*
Married (sep.)	0.387***	0.327**	0.281*	0.298**	0.140	0.125
	(0.144)	(0.146)	(0.151)	(0.151)	(0.152)	(0.206)
Pacs (comm.)	0.286	0.125	0.182	0.263	0.364	0.188
	(0.261)	(0.263)	(0.264)	(0.267)	(0.270)	(0.366)
Pacs (Sep.)	0.259*	-0.012	-0.009	-0.006	-0.121	0.452**
	(0.140)	(0.150)	(0.152)	(0.152)	(0.153)	(0.208)
Demographics		X	X	X	X	X
Professional			X	X	X	X
Bequest				X	X	X
Observations	1783	1783	1783	1783	1783	1783
R^2	0.007	0.037	0.045	0.053	0.090	0.053

Data: *Patrimoine* surveys 2014–2015 and 2017–2018.
Note: Standard errors in parentheses. * p<.1, ** p<.05, *** p<.01. Demographic characteristics include: male partner's birth cohort, age difference between partners, year of couple formation, size of residence city, citizenship of both partners, and past marriage. Professional characteristics include: educational attainment of both partners, 2015 household income (in log), changes in household income (2015–2018), at least one partner is self-employed in 2015, change in self-employment (2015–2018), at least one partner is not working full-time in 2015, and change in full-time employment (2015–2018). Bequest characteristics include: dummy variable for receiving a bequest or a gift (2015–2018), dummy variable for giving an inter vivos gift (2015–2018), number of children in the household in 2015, binary for a decrease in the number of children (2015–2018), and binary for an increase in number of children (2015–2018). Wealth is expressed in 1,000 euros (2015), IHS transformed, and constructed as follows. Net wealth: the sum of all the couple's assets minus liabilities (excluding business assets). Net housing wealth: the sum of all the couple's housing assets minus housing debt. Net financial wealth: the sum of all the couple's financial assets minus non-housing debt.

Table B.14: Marital status and changes in female partner's share of household net wealth, sample including couples transitioning across marital status (2015–2018)

	(1)<br>Female<br>share	(2)<br>Female<br>share	(3)<br>Female<br>share	(4)<br>Female<br>share	(5)<br>Female<br>share	(6)<br>Female<br>share
Cohab.	-0.004	-0.012	-0.012	-0.009	0.078***	-0.072***
	(0.011)	(0.013)	(0.013)	(0.013)	(0.014)	(0.015)
Married (comm.)	*Ref.*	*Ref.*	*Ref.*	*Ref.*	*Ref.*	*Ref.*
Married (sep.)	0.021	0.022	0.021	0.021	0.060***	-0.043*
	(0.017)	(0.017)	(0.018)	(0.018)	(0.018)	(0.023)
PACS (sep.)	0.001	-0.008	-0.003	-0.001	0.056***	-0.069***
	(0.016)	(0.018)	(0.018)	(0.018)	(0.019)	(0.021)
F richer than M					-0.122***	
					(0.023)	
Cohab. × F richer than M					-0.138***	
					(0.029)	
Married (sep.) × F richer than M					-0.098**	
					(0.042)	
PACS (sep.) × F richer than M					-0.117***	
					(0.039)	
F inc. sh. > F W. sh.						0.053***
						(0.012)
Cohab. × F inc. sh. > F W. sh.						0.160***
						(0.022)
Married (sep.) × F inc. sh. > F W. sh.						0.112***
						(0.032)
PACS (sep.) × F inc. sh. > F W. sh.						0.171***
						(0.032)
Constant	0.002	0.002	0.057	0.052	0.025	-0.024
	(0.006)	(0.023)	(0.048)	(0.049)	(0.045)	(0.047)
Demographics		X	X	X	X	X
Professional			X	X	X	X
Bequest				X	X	X
Wealth gap between spouses					X	
Female wealth/income comparison						X
Observations	1722	1722	1722	1722	1722	1722
R^2	0.001	0.017	0.035	0.038	0.191	0.152

Data: *Patrimoine* surveys 2014–2015 and 2017–2018.
Note: Standard errors in parentheses. * p<.1, ** p<.05, *** p<.01. Demographic characteristics include: male partner's birth cohort, age difference between partners, year of couple formation, size of residence city, citizenship of both partners, and past marriage. Professional characteristics include: educational attainment of both partners, 2015 household income (in log), changes in household income (2015–2018), at least one partner is self-employed in 2015, change in self-employment (2015–2018), at least one partner is not working full-time in 2015, and change in full-time employment (2015–2018). Bequest characteristics include: dummy variable for receiving a bequest or a gift (2015–2018), dummy variable for giving an inter vivos gift (2015–2018), number of children in the household in 2015, binary for a decrease in the number of children (2015–2018), and binary for an increase in number of children (2015–2018). Female partner's share is her share of the net household wealth. Net wealth is constructed as the sum of all the couple's assets minus liabilities (excluding business assets).

Table B.15: Legal status and net wealth accumulation, controlling for 2015 level of wealth

	(1) Net wealth	(2) Net wealth	(3) Net housing wealth	(4) Net financial wealth
Unregist. cohab.	0.027	-0.109	0.099	0.107
	(0.100)	(0.114)	(0.114)	(0.159)
PACS	0.071	-0.092	-0.058	0.400**
	(0.127)	(0.138)	(0.138)	(0.192)
Married	*Ref.*	*Ref.*	*Ref.*	*Ref.*
Net wealth in 2015	-0.001***	-0.001***		
	(0.000)	(0.000)		
Net housing wealth in 2015			-0.002***	
			(0.000)	
Net financial wealth in 2015				-0.002***
				(0.000)
Demographics		X	X	X
Professional		X	X	X
Bequest		X	X	X
Observations	1666	1666	1666	1666
R^2	0.028	0.070	0.130	0.086

Data: *Patrimoine* surveys 2014–2015 and 2017–2018.
Note: Standard errors in parentheses. * p<.1, ** p<.05, *** p<.01. Demographic characteristics include: male partner's birth cohort, age difference between partners, year of couple formation, size of residence city, citizenship of both partners, and past marriage. Professional characteristics include: educational attainment of both partners, 2015 household income (in log), changes in household income (2015–2018), at least one partner is self-employed in 2015, change in self-employment (2015–2018), at least one partner is not working full-time in 2015, and change in full-time employment (2015–2018). Bequest characteristics include: dummy variable for receiving a bequest or a gift (2015–2018), dummy variable for giving an inter vivos gift (2015–2018), number of children in the household in 2015, binary for a decrease in the number of children (2015–2018), and binary for an increase in number of children (2015–2018). Wealth is expressed in 1,000 euros (2015), IHS transformed, and constructed as follows. Net wealth: the sum of all the couple's assets minus liabilities (excluding business assets). Net housing wealth: the sum of all the couple's housing assets minus housing debt. Net financial wealth: the sum of all the couple's financial assets minus non-housing debt.

Table B.16: Property regime and net wealth accumulation, controlling for 2015 level of wealth

	(1) Net wealth	(2) Net wealth	(3) Net housing wealth	(4) Net financial wealth
Sep. property	0.202**	0.075	0.129	0.211*
	(0.078)	(0.091)	(0.091)	(0.126)
Net wealth in 2015	-0.001***	-0.001***		
	(0.000)	(0.000)		
Net housing wealth in 2015			-0.002***	
			(0.000)	
Net financial wealth in 2015				-0.002***
				(0.000)
Demographics		X	X	X
Professional		X	X	X
Bequest		X	X	X
Observations	1666	1666	1666	1666
R^2	0.032	0.069	0.130	0.085

Data: *Patrimoine* surveys 2014–2015 and 2017–2018.
Note: Standard errors in parentheses. * p<.1, ** p<.05, *** p<.01. Demographic characteristics include: male partner's birth cohort, age difference between partners, year of couple formation, size of residence city, citizenship of both partners, and past marriage. Professional characteristics include: educational attainment of both partners, 2015 household income (in log), changes in household income (2015–2018), at least one partner is self-employed in 2015, change in self-employment (2015–2018), at least one partner is not working full-time in 2015, and change in full-time employment (2015–2018). Bequest characteristics include: dummy variable for receiving a bequest or a gift (2015–2018), dummy variable for giving an inter vivos gift (2015–2018), number of children in the household in 2015, binary for a decrease in the number of children (2015–2018), and binary for an increase in number of children (2015–2018). Wealth is expressed in 1,000 euros (2015), IHS transformed, and constructed as follows. Net wealth: the sum of all the couple's assets minus liabilities (excluding business assets). Net housing wealth: the sum of all the couple's housing assets minus housing debt. Net financial wealth: the sum of all the couple's financial assets minus non-housing debt.

Table B.17: Marital status and net wealth accumulation, controlling for 2015 level of wealth

	(1)<br>Net<br>wealth	(2)<br>Net<br>wealth	(3)<br>Net housing<br>wealth	(4)<br>Net financial<br>wealth
Cohab.	0.080	-0.056	0.136	0.135
	(0.100)	(0.116)	(0.116)	(0.161)
Married (comm.)	*Ref.*	*Ref.*	*Ref.*	*Ref.*
Married (sep.)	0.559***	0.416***	0.330**	0.182
	(0.140)	(0.146)	(0.147)	(0.203)
PACS (comm.)	0.154	0.103	0.287	0.266
	(0.266)	(0.274)	(0.275)	(0.383)
PACS (sep.)	0.117	-0.067	-0.090	0.471**
	(0.140)	(0.153)	(0.154)	(0.214)
Net wealth in 2015	-0.001***	-0.001***		
	(0.000)	(0.000)		
Net housing wealth in 2015			-0.002***	
			(0.000)	
Net financial wealth in 2015				-0.002***
				(0.000)
Demographics		X	X	X
Professional		X	X	X
Bequest		X	X	X
Observations	1666	1666	1666	1666
R^2	0.038	0.074	0.134	0.087

Data: *Patrimoine* surveys 2014–2015 and 2017–2018.
Note: Standard errors in parentheses. * p<.1, ** p<.05, *** p<.01. Demographic characteristics include: male partner's birth cohort, age difference between partners, year of couple formation, size of residence city, citizenship of both partners, and past marriage. Professional characteristics include: educational attainment of both partners, 2015 household income (in log), changes in household income (2015–2018), at least one partner is self-employed in 2015, change in self-employment (2015–2018), at least one partner is not working full-time in 2015, and change in full-time employment (2015–2018). Bequest characteristics include: dummy variable for receiving a bequest or a gift (2015–2018), dummy variable for giving an inter vivos gift (2015–2018), number of children in the household in 2015, binary for a decrease in the number of children (2015–2018), and binary for an increase in number of children (2015–2018). Wealth is expressed in 1,000 euros (2015), IHS transformed, and constructed as follows. Net wealth: the sum of all the couple's assets minus liabilities (excluding business assets). Net housing wealth: the sum of all the couple's housing assets minus housing debt. Net financial wealth: the sum of all the couple's financial assets minus non-housing debt.
